# Effects of Pacing Site and Stimulation History on Alternans Dynamics and the Development of Complex Spatiotemporal Patterns in Cardiac Tissue

**DOI:** 10.3389/fphys.2013.00071

**Published:** 2013-04-19

**Authors:** Alessio Gizzi, Elizabeth M. Cherry, Robert F. Gilmour, Stefan Luther, Simonetta Filippi, Flavio H. Fenton

**Affiliations:** ^1^Non-linear Physics and Mathematical Modeling Laboratory, University Campus Bio-Medico of RomeRome, Italy; ^2^School of Mathematical Sciences, Rochester Institute of TechnologyRochester, NY, USA; ^3^University of Prince Edward IslandCharlottetown, PE, Canada; ^4^Max Planck Institute for Dynamics and Self-OrganizationGottingen, Germany; ^5^School of Physics, Georgia Institute of TechnologyAtlanta, GA, USA

**Keywords:** cardiac alternans, optical mapping, cardiac electrophysiology, ventricular arrhythmias, spatiotemporal patterns

## Abstract

Alternans of action potential duration has been associated with T wave alternans and the development of arrhythmias because it produces large gradients of repolarization. However, little is known about alternans dynamics in large mammalian hearts. Using optical mapping to record electrical activations simultaneously from the epicardium and endocardium of 9 canine right ventricles, we demonstrate novel arrhythmogenic complex spatiotemporal dynamics. (i) Alternans predominantly develops first on the endocardium. (ii) The postulated simple progression from normal rhythm to concordant to discordant alternans is not always observed; concordant alternans can develop from discordant alternans as the pacing period is decreased. (iii) In contrast to smaller tissue preparations, multiple stationary nodal lines may exist and need not be perpendicular to the pacing site or to each other. (iv) Alternans has fully three-dimensional dynamics and the epicardium and endocardium can show significantly different dynamics: multiple nodal surfaces can be transmural or intramural and can form concave/convex surfaces resulting in islands of discordant alternans. (v) The complex spatiotemporal patterns observed during alternans are very sensitive to both the site of stimulation and the stimulation history. Alternans in canine ventricles not only exhibit larger amplitudes and persist for longer cycle length regimes compared to those found in smaller mammalian hearts, but also show novel dynamics not previously described that enhance dispersion and show high sensitivity to initial conditions. This indicates some underlying predisposition to chaos and can help to guide the design of new drugs and devices controlling and preventing arrhythmic events.

## 1 Introduction

**Figure 1:**
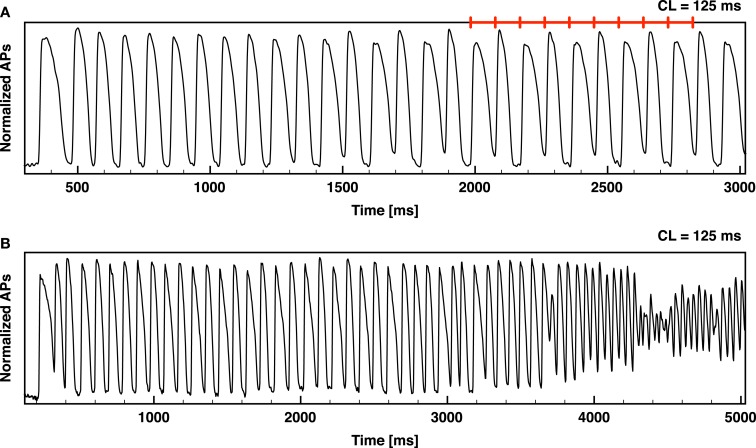
**Alternans development leading to ventricular fibrillation**. Optical signal from an RV canine preparation showing alternans development and alternans leading to ventricular fibrillation (VF). **(A)** RV paced from rest at a constant CL of 125 ms (denoted by the red segments); sustained alternans develops after a few beats with APD75 alternating between 83 and 42 ms. **(B)** RV paced from rest at a constant CL of 125 ms resulting in alternans and then VF.

It is well known that increased dispersion of repolarization can lead to large variations in refractory period and conduction velocity that can, in turn, induce fibrillation (Han and Moe, 1964; Kuo et al., 1983). Over the last few years, spatiotemporal dispersion of the action potential duration (APD) at the cellular and tissue levels have been linked experimentally (Pastore et al., 1999; Walker et al., 2003; Walker and Rosenbaum, 2005; de Diego et al., 2008; Weinberg et al., 2010) and theoretically (Qu et al., 2000; Watanabe et al., 2001) to T wave alternans (TWA) and to its clinical utility in stratifying risk for sudden cardiac death, even at the micro-volt level (Rosenbaum et al., 1994; Verrier et al., 2011). Dispersion of APD has been shown to develop in ventricular tissue during fast pacing (Pastore et al., 1999; Walker and Rosenbaum, 2005; Choi et al., 2007; Hayashi et al., 2007; Mitrea et al., 2009; Ziv et al., 2009), where a period-doubling bifurcation (Chialvo et al., 1990; Hall et al., 1999; Mitrea et al., 2009) develops, resulting in alternation of the action potential between long and short durations, even while the period remains constant, as shown in Figure 1A. Spatially this dynamics can develop into discordant alternans, where part of the tissue responds with a short APD, while another responds with a long APD (Pastore et al., 1999; Qu et al., 2000; Watanabe et al., 2001). This behavior can result in very large local gradients of repolarization during consecutive beats that can cause conduction block (Watanabe et al., 2001; Fox et al., 2002) and the initiation of arrhythmias (Choi and Salama, 2000; Cherry and Fenton, 2008) such as tachycardia and fibrillation, as shown in Figure 1B. Although there have been many studies quantifying the dynamics of alternans in space, they have all been conducted in monolayers (Shiferaw et al., 2005; Bien et al., 2006; Jia et al., 2007; Kim et al., 2007; de Diego et al., 2008; Weinberg et al., 2010) or small mammalian hearts (Pastore et al., 1999; Choi and Salama, 2000; Pastore and Rosenbaum, 2000; Walker et al., 2003; Hayashi et al., 2007; Mironov et al., 2008; Myles et al., 2008, 2011; Hsieh et al., 2009; Ziv et al., 2009).

Consequently, little is known about the spatiotemporal dynamics of alternans in large mammalian hearts. The purpose of this study is to characterize the dynamics of alternans in canine ventricular muscle, where not only its cell physiology is similar to that of human cells (Kääb et al., 1996; Li et al., 1998) but the larger tissue size allows us to investigate the development of alternans in longer and thicker domains where alternans can develop in a fully three-dimensional configuration. Furthermore, alternans in canine tissue has been shown to develop readily at normal physiological temperatures for pacing cycle lengths below 250 ms and alternans can be sustained for cycle lengths as short as 100 ms (Koller et al., 1998; Cherry and Fenton, 2007). Therefore, by using canine tissue we are able to study alternans in large preparations at physiological temperatures for a large range of pacing cycle lengths, which is not possible in smaller mammalian hearts.

We present here the results from 9 canine right ventricular (RV) preparations in which a total of 1362 epicardial/endocardial optical mapping recordings were performed and analyzed to determine how concordant and discordant alternans develops and evolve over time. We investigated the development of alternans simultaneously on the epicardium and endocardium at normal body temperature (37°C) as a function of pacing cycle length, pacing site, and pacing history. The results suggest multiple not previously described dynamical paths leading to ventricular tachyarrhythmias.

## 2 Materials and Methods

### 2.1 Ethical approval of the procedure for the specimen preparation

All experimental procedures were approved by the Institutional Animal Care and Use Committee of the Center for Animal Resources and Education at Cornell University. The tissue preparation has been previously described (Luther et al., 2011). Briefly, adult beagle dogs of either sex (*n* = 9), age 1–4 years, were anesthetized with Fatal-Plus (390 mg/mL pentobarbital sodium; Vortex Pharmaceuticals; 86 mg/kg IV), and their hearts were rapidly excised via a left thoracotomy and placed in cold, aerated (95% O25% CO_2_) normal Tyrode solution containing (in mmol L^−1^): 124 NaCl, 4.0 KCl, 24 NaHCO3, 0.9 NaH2PO4, 2.0 CaCl2, 0.7 MgCl2, and 5.5 glucose, adjusted to pH 7.4 with NaOH. The right coronary artery was cannulated with polyethylene tubing, and the right ventricular myocardium was excised and suspended in a heated transparent tissue Plexiglas chamber. It was both perfused (perfusion pressure of 50–80 mmHg, flow rate was 25 mL/min) and superfused with normal Tyrode solution by that artery. After 15–20 min of equilibration at physiological temperature (37.0 ± 0.5°C), the preparation was stained with the voltage-sensitive dye Di-4-ANEPPS (10 μmol/L bolus). Blebbistatin (10 μmol/L constant infusion over 30–40 min) was added to prevent motion artifact.

### 2.2 Optical mapping

The optical mapping setup has been described previously (Cherry and Fenton, 2008; Fenton et al., 2009). Briefly, illumination was provided by high-performance light-emitting diodes (Luxeon III star, LXHL-FM3C, wavelength 530 nm), nine for the top view and nine for the bottom view, driven by a low-noise constant-current source. The illumination efficiency was significantly enhanced by collimator lenses (Luxeon, LXHL-NX05). The epicardium and endocardium were imaged simultaneously using two synchronized cameras. The fluorescence emission light was collected for each camera by a Navitar lens (DO-2595, focal length 25 mm, F# 0.95), passed through a long-pass filter (<610 nm), and imaged by a 128 × 128 back-illuminated electron-multiplied charge-coupled device array (Photometrics Cascade 128+) with a high quantum efficiency (peak QE > 90%). The signal was digitized with a 16-bit analog/digital converter at a frame rate of 511 Hz (full frame, 128 × 128 pixels) with a spatial resolution of 600 μm per pixel for a grid size of 7.7 cm × 7.7 cm. The peripheral component interconnect interface provided high-bandwidth uninterrupted data transfer to the host computer. Throughout the duration of the experiment instantaneous signals from one pixel of the epicardium and endocardium were displayed on a computer screen to assess signal quality and capture during pacing. Data were analyzed with a custom built Java program with interactive tools for visualization, segmentation, filtering, and saving.

### 2.3 Stimulation protocol

We applied two different temporal pacing protocols: (1) pacing-down protocol. Twice diastolic threshold current pulses were applied with the pacing cycle length (CL) starting from 550 ms and decreasing in 50 ms decrements until reaching 250 ms, after which the CL was shortened in 10 ms decrements until capture was lost or VF was induced. At each CL, pacing was applied for at least 1 min before recording to ensure that steady state was reached, then recordings were made for 10–15 s at each CL. (2) Quiescent protocol. Twice diastolic threshold current pulses were applied at constant CLs of 220, 200, 160, 150, 110, or 100 ms for at least 1 min to tissue that was previously at rest (quiescent for at least 5 min). We applied two different spatial pacing protocols for each of the two temporal protocols. In one case, we maintained the pacing electrode fixed at the same anatomical position on the endocardium. In the second case, we moved the pacing electrode between four orthogonal anatomical positions on the endocardium: base, anterior, apex, and posterior (see Figure S1 in the Supplementary Material). Whenever VF was induced, it was terminated using LEAP (Low Energy Anti-Fibrillation Pacing) (Fenton et al., 2009; Luther et al., 2011).

### 2.4 Data analysis

We analyzed 1362 recordings (with duration between 5000 and 15,000 ms) using custom built interactive Java program. Data were processed to remove signal drift and fluorescence noise; normalization was conducted on a pixel-by-pixel basis and the time averages of length 7 (3 forward and 3 backward) and weighted Gaussian space averages (8 neighbor pixels) of the signal were performed. Pixels not visualizing the tissue were removed by a user defined mask. Optical APDs were measured at 75% repolarization threshold (APD75) obtained using standard linear interpolation (Glukhov et al., 2010).

To determine the temporal distribution of AP alternans across the mapped field, we computed the difference between two consecutive APDs defined as:

(1) $$\begin{array}{llll} & \Delta \text{APD}{\left(x,y\right)}_{n}=\text{APD}{\left(x,y\right)}_{n+1}-\text{APD}{\left(x,y\right)}_{n} & & \\ & \;\to \left\{\begin{array}{cc} |\Delta \text{APD}{\left(x,y\right)}_{n}|>2\text{ms}\; & \;\text{Alternans (RED - BLUE)}\\ |\Delta \text{APD}{\left(x,y\right)}_{n}|\leq 2\text{ms}\; & \;\text{Nodal line (WHITE)}\\ \; \end{array}\right. & \end{array}$$

where n denotes the beat number and APD(x, y) is the duration of the action potential at a pixel in position (*x*, *y*) in the 2D mapped field. Due to our temporal resolution, 2 ms, tissue was defined as non-alternating when Δ*APD*(*x*, *y*, *n*) was greater than −2 and less than 2 ms, and as alternating otherwise. The phase of alternans was negative for short-long APD sequences (blue scale) and positive for long-short APD sequences (red scale). Nodal lines were defined as areas in which the amplitude of alternans was below the described threshold that separated out-of-phase regions of discordant alternans. Two-dimensional alternans maps were constructed to explore the spatial distribution of the amplitude and phase of the alternans. The onset of alternans [both concordant alternans (CA) or discordant alternans (DA)] was defined separately between endocardial and epicardial surfaces as the CL at which at least 5% of the surface pixels displayed APD alternans. The local CV was only measured in the direction of the propagating wave using the distributions of activation maps for spatial regions of 7 × 7 or 8 × 8 pixels.

## 3 Results

Alternans in RV canine preparations was quantified using simultaneous recordings of the epicardium and endocardium and the results are presented in eight sections. In the following we will refer to a fixed color code for alternans maps with 10 intervals of 5 ms: positive intervals (+[0:50] ms) will be identified from red to yellow, negative intervals (−[0:50] ms) from blue to green. In the first section we investigate whether alternans appears in a way similar to that of previous studies using smaller hearts and monolayers, after which we perform (section two) the first quantitative study of differences in alternans properties of the epicardium and endocardium by characterizing the onset and evolution of alternans on the two surfaces as a function of pacing frequency. In the third section, we infer the dynamics of intramural nodal surfaces and show the high level of complexity that can develop even in the small thickness of the RV. In the fourth section we show that as cycle length is decreased in the larger tissues of canine ventricles, it is possible to convert from discordant alternans back to concordant and then again to discordant alternans as the cycle length decreases, which can lead to separate regions of alternans with various degrees of complexity as a function of cycle length. This complex spatiotemporal dynamics is further discussed in section five, where we show that higher-order rhythms such as 4:4 and 8:8 can appear in tissue. In sections six and seven we quantify the effects of pacing position and timing on the onset and dynamics of alternans. Finally, in section eight we show the large variability in patterns of discordant alternans that can be obtained in tissue even with what appear to be the same initial conditions.

### 3.1 Concordant and discordant alternans in canine RV tissue

**Figure 2:**
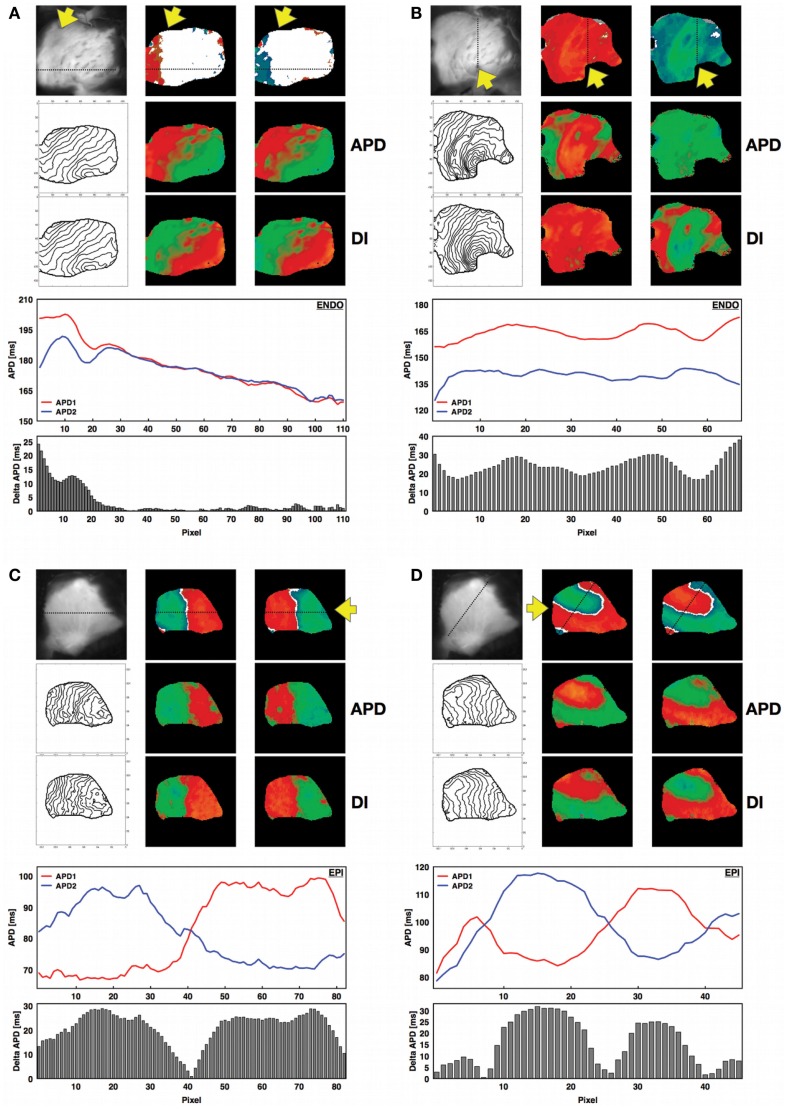
**Concordant and discordant alternans in canine RV preparations**. For all four panels a photograph of the corresponding RV structure is shown next to the spatial alternans maps for two consecutive beats after reaching steady state (see Figure S2 in the Supplementary Material). The yellow arrow indicates the site of stimulation on the endocardium. The APD and DI spatial distributions are shown below for the same beats, together with the corresponding isochrone maps on the left. In addition, the APDs calculated along the line indicated on the alternans maps are shown for the two consecutive beats at steady state (red: first beat; blue: second beat), along with the magnitude of alternans (difference in consecutive APDs) along the same line as a histogram for each pixel. **(A)** Regional concordant alternans (beats 18–19, CL = 400 ms, APD = [143–212] ms, DI = [176–237] ms, endocardium, main axis 7 cm), **(B)** global concordant alternans (beats 13–14, CL = 220 ms, APD = [114–173] ms, DI = [63–136] ms, endocardium, main axis 7 cm), **(C)** global discordant alternans with one node (beats 19–20, CL = 110 ms, APD = [63–107] ms, DI = [26–74] ms, epicardium, main axis 6 cm), **(D)** global discordant alternans with multiple (three) nodes (beats 37–38, CL = 150 ms, APD = [70–124] ms, DI = [25–82] ms, epicardium, main axis 6 cm). Figure S2 in the Supplementary Material shows nodal patterns for all the beats. **(A,B)** Recordings are from endocardium, while **(C,D)** are from epicardium. Similar behavior was observed on both surfaces.

The canine RV preparations used for this study exhibited the same basic patterns of concordant and discordant alternans that have been observed in other cardiac tissue preparations, where either the full tissue is alternating with short and long action potentials (CA) or there is a nodal line separating regions of long and short (DA). However, we found that other types of patterns also occurred (Figure 2). When concordant alternans first developed, it did not occupy the entire tissue at the same time; as shown in Figure 2A, when alternans first appeared it was always a partial section with only one region exhibiting 2:2 behavior while the rest remained in 1:1. Only 50% of the preparations in the epicardium (78% in the endocardium) had full tissue CA present (Figure 2B) before DA developed. Cardiac tissue also readily showed discordant alternans with one or more nodal lines where the APD remained constant on consecutive beats. Figure 2C shows an example of discordant alternans observed with a single nodal line and Figure 2D shows a more complicated pattern with three regions of alternans separated by two nodal lines. Although one of the regions in Figure 2D is relatively small, the APDs from successive beats along a diagonal line across the tissue clearly show the large difference in alternans magnitudes and phases between the discordant alternans regions separated by three nodes (two from multiple crossings of the same nodal line). Note that in all cases, the alternans map is shown for two consecutive beats to demonstrate that the patterns were stable and that steady state was reached. (See also Figure S2 in Supplementary Material which shows the maps for each paced beat leading to steady states for the four examples shown in Figure 2).

### 3.2 Epicardial and endocardial dynamics during alternans

**Figure 3:**
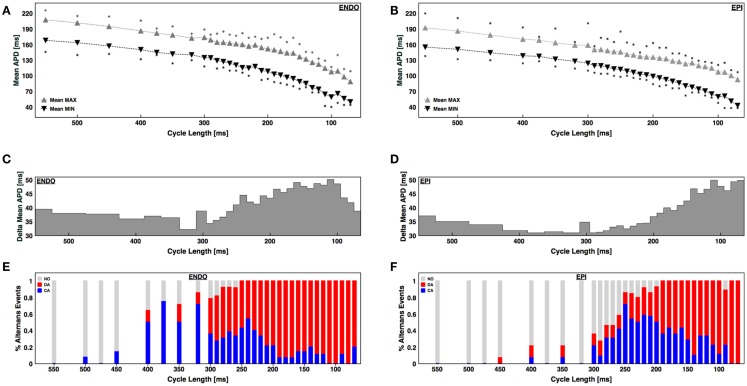
**APD dispersion and alternans development for the endocardium and epicardium**. **(A,B)** Mean maximum APD (gray triangles) and mean minimum APD (black triangles) within the tissue for each CL for **(A)** endocardium and **(B)** epicardium. The squares indicate the maximum and minimum APDs from all preparations. **(C,D)** Difference in maximum and minimum APDs as a function of CL for **(C)** endocardium and **(D)** epicardium. **(E,F)** Percentage of cases with no alternans (gray), concordant alternans (red), and discordant alternans (blue) as a function of CL for endocardium **(E)** and epicardium **(F)**.

To quantify regional differences in alternans properties, we compared the cycle lengths for which alternans was present on the epicardial and endocardial surfaces in 14 different sets of recordings (720 cases) taken from 6 canine RV preparations. A large range of cycle lengths ranging from 500 ms down to conduction block or VF initiation was used. Even before alternans was present, there was a noticeable spatial dispersion of APD of about 30–40 ms on both surfaces, as measured by the difference between the maximum and minimum APDs during steady state (Figures 3A,B). As the CL was decreased and alternans appeared, the dispersion between maximum and minimum APD grew, but only by an additional 10–15 ms; however, it is important to note that before alternans the variations between APDs were distributed through space whereas during alternans the large variation occurred locally from one beat to the next. Interestingly, as the BCL was decreased, the dispersion magnitude decreased slightly for both epicardium and endocardium before increasing again during alternans (Figures 3C,D). In addition, the CL corresponding to the onset of alternans, as well as the likelihood of developing concordant and discordant alternans at a given CL, differed between the endocardium and epicardium. Figures 3E,F shows the percentage of experiments without alternans (gray), with concordant alternans (red), and with discordant alternans (blue) for each CL. Discordant alternans was likely to occur on the endocardium for CLs below 300 ms, with the probability increasing for CLs below 240 ms. In contrast, discordant alternans was much less likely to occur on the epicardium for CLs longer than 200 ms (648 recordings, 324 on ENDO and 324 on EPI). Compared to previous studies in other mammalian hearts, we found the range and magnitudes of alternans to be much larger in RV canine tissue at physiological temperatures (Figure 3), where alternans appeared in one case at a CL as large as 500 ms and continued to up to 80 ms before 2:1 conduction block occurred. The largest CL for which at least 20% of the preparations showed alternans was 400 ms and the maximum alternans amplitude was observed during discordant alternans and in the range between 45 and 50 ms (Figures 3C,D).

### 3.3 From nodal lines to nodal surfaces

**Figure 4:**
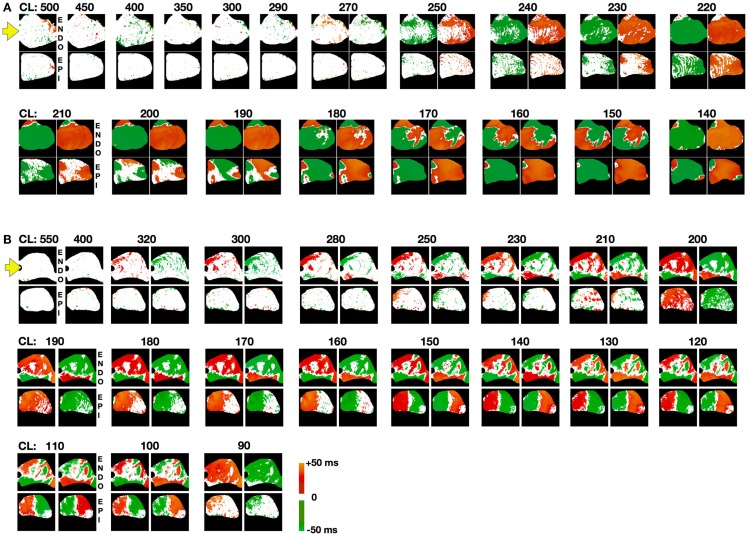
**Steady state spatial alternans patterns as a function of CL**. Spatial alternans patterns from endocardial (top) and epicardial (bottom) RV preparations (main axis 7 cm). Representative examples from two different preparations are shown in **(A,B)**. Maps from two consecutive beats are shown for each case where alternans was present. Note that discordant alternans developed at longer CLs on the endocardium than the epicardium and that there was no apparent correlation between the two surfaces. Yellow arrow indicates pacing site. Color code refers to alternans maps amplitude with 10 intervals of 5 ms: no alternating regions (white), positive intervals (+[0:50] ms; red to yellow), negative intervals (−[0:50] ms) (blue to green). Steady state was reached for each CL by pacing for 60 s followed by recording for an additional 10–15 s.

To further quantify transmural differences in alternans properties, we compared alternans maps from simultaneous epicardial and endocardial surfaces recordings (834 recordings) taken from 6 canine RV preparations paced at cycle lengths ranging from 500 ms down to conduction block or VF initiation. By comparing the maps from the two surfaces, it was possible to gain information about the development and dynamics of alternans intramurally, particularly regarding the three-dimensional nodal lines or nodal surfaces. Nodal line patterns on the endocardium and epicardium usually differed significantly, which suggested that intramural nodal surfaces must have had a complex topology. Figure 4 shows the steady state endocardial and epicardial alternans maps for consecutive beats over a range of cycle lengths to illustrate the complex evolution of nodal surfaces as the CL was decreased for two representative preparations. Alternans developed at longer CLs for the endocardium than for the epicardium and the alternans patterns often were more complicated for the endocardium where 79% of all DA cases had more than one nodal line vs. the epicardium with 57%. As shown in Figure 2D, it was possible for multiple nodal lines to develop and even to form islands of alternans resulting from nodal lines forming a loop. It was evident from the nodal line distributions at the surfaces of the epicardium and endocardium shown in Figure 4A (for CLs near 190 ms) and Figure 4B (for CLs between 160 and 120 ms) that intramural nodal surfaces were not simply perpendicular to the thickness of the tissue. In fact, given the differences between the nodal line patterns on the two surfaces, it was clear that the nodal surfaces through the ventricular wall could be quite complicated. Qualitatively similar nodal line patterns between the epicardium and endocardium like those shown in Figure 4A for a CL of 140 ms, which would strongly suggest that the intramural pattern of the nodal surface was the same (mostly perpendicular to the thickness), were rarely observed, occurring only on 8% of all DA cases.

### 3.4 Transitions between concordant and discordant alternans

**Figure 5:**
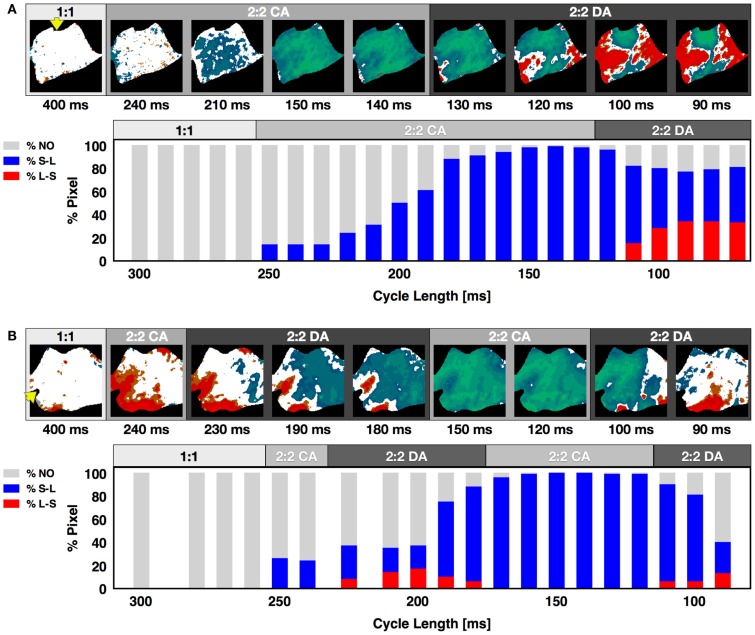
**Alternans transition between concordant and discordant rhythms**. Representative examples of the standard and extended alternans transitions between concordant and discordant alternans as CL was decreased. **(A)** Standard transition from no alternans to concordant alternans to discordant alternans. Alternans maps for selected CLs are shown within each of the three regions. The lower plot shows the percentage of pixels in a given state at each CL. Gray represents pixels with no alternans, blue pixels alternating short-long, and red pixels alternating long-short; the presence of both red and blue colors indicates discordant alternans. **(B)** Extended transition from no alternans to concordant alternans to discordant alternans, then back to concordant alternans and discordant alternans. Yellow arrow indicates pacing site. The main axis is 7 cm.

Although alternans has been observed previously both experimentally (Rosenbaum et al., 1994; Pastore et al., 1999; Walker et al., 2003; de Diego et al., 2008; Weinberg et al., 2010) and theoretically (Qu et al., 2000; Watanabe et al., 2001) to develop first as concordant alternans and then progress to discordant alternans as the pacing CL is decreased until conduction block is reached or fibrillation is initiated, we found that this standard alternans transition was not always the case. In fact, it was possible for concordant alternans to recur at shorter CLs after a range of CLs where discordant alternans was present, as can be seen in Figure 4B for a CL of 90 ms. In some cases a second discordant alternans region followed a second concordant alternans region (what we call the extended alternans transition). Figure 5 shows examples of the standard and the extended transitions. We observed this extended transition in 21% of the cases on the endocardium and 14% on the epicardium. The standard transitions are illustrated in Figure 5A, where alternans maps for selected CLs display the three different behaviors (no alternans, concordant alternans and discordant alternans) observed before reaching conduction block or fibrillation. The histogram distribution shows that alternans did not occur in this preparation until the appearance of concordant alternans at a CL of 300 ms, followed by the development of fully concordant alternans at 150 ms and discordant alternans between 110 and 70 ms, ending with conduction block at a CL of 60 ms. Figure 5B shows the extended transition, where discordant alternans reverts to concordant alternans as the CL is decreased. In this case, CA developed twice, for CLs between 300 and 225 ms and again between 170 and 120 ms, with DA also occurring twice, for CLs between 225 and 170 ms and below 120 ms.

### 3.5 Alternans and higher-order rhythms (N:N dynamics)

**Figure 6:**
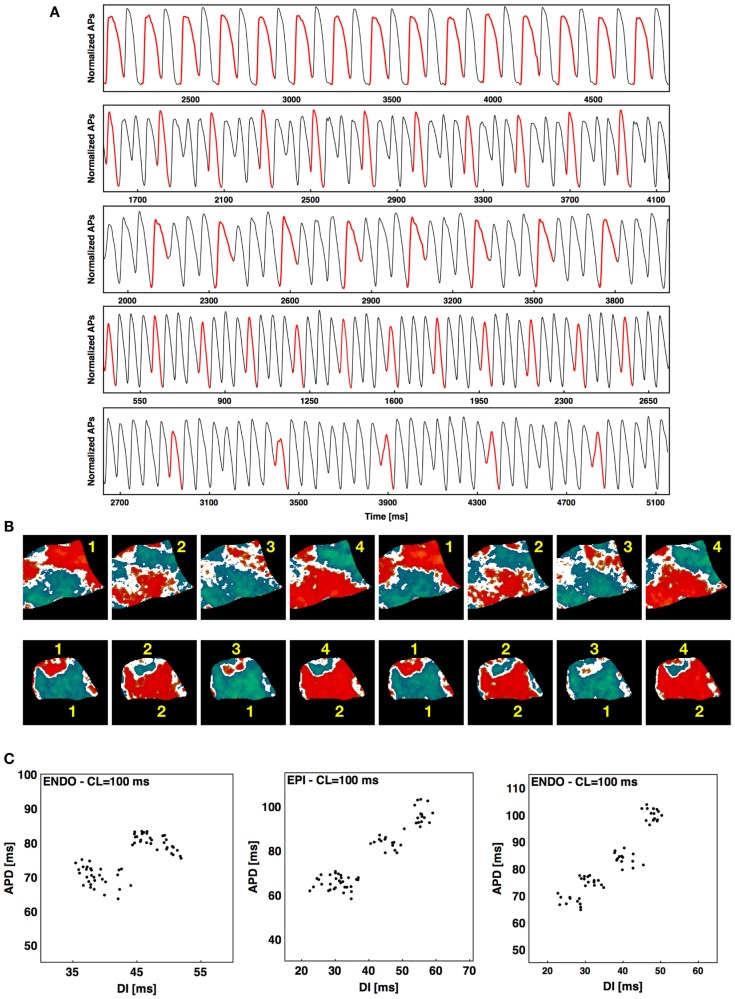
**Higher-order rhythms**. **(A)** From top to bottom 2:2, 4:4, 4:4, 4:4, and 8:8 rhythms. The first AP of each rhythm is shown in red to facilitate visualization. **(B)** Top: alternans map of an endocardial preparation (main axis 7 cm) with a 4:4 sequence; bottom: alternans map of an epicardial preparation (main axis 6 cm) with mixed rhythms (4:4 at the top and 2:2 at the bottom). Two consecutive sequences are shown for both cases; see Figure S3 in Supplementary Material for the full sequence. **(C)** Plots of APD vs. DI from one pixel over time during steady state showing an example of 2:2 rhythm and two examples of 4:4 rhythm, with the first 4:4 a degenerate case. See text and Figure S4 in Supplementary Material for details.

Although most often during rapid pacing-induced alternans a period-two (2:2) response was observed (as seen in Figure 1), in about 2% of the cases higher-order periodicities were observed. For example, a long-short-long-short pattern in which two distinct long and two distinct short APDs occurred before the sequence repeated itself was observed 1.3% of the time and constituted a stable 4:4 pattern. Figure 6A shows a series of higher-order rhythms, beginning with a standard 2:2 rhythm in the first panel, followed by three examples of a 4:4 rhythm and an example of an 8:8 rhythm, which was observed considerably less frequently at 0.65% of the episodes. These rhythms sometimes occurred globally throughout the tissue, as shown in the upper panel of Figure 6B for a 4:4 case (see Figure S3A in Supplementary Material for the full sequence), but mostly they occurred only in local regions of the tissue while the rest of the tissue followed a standard 2:2 rhythm. The lower panel of Figure 6B shows an example of this regional variation in rhythm, where a 4:4 rhythm occurred in the upper part of the tissue and the lower portion followed a standard 2:2 rhythm (see Figure S3B in Supplementary Material for full sequence). Plotting APD as a function of DI for a single pixel over time provides an alternative means for visualizing higher-order rhythms when the change in morphology of the action potential is not easily identified. Figure 6C shows representative plots of APD as a function of DI for a 2:2 case and two 4:4 cases. Although the central plot appears to indicate a much higher 3:3 order rhythm, such rhythms do not occur as part of a period-doubling cascade, and a careful analysis shows that this scenario is a degenerate case of a 4:4 rhythm in which the two short APDs are nearly identical (see Figure S4 in Supplementary Material), producing a pattern that resembled a 3:3 rhythm.

### 3.6 Effect of pacing site on alternans initiation and nodal line patterns

**Figure 7:**
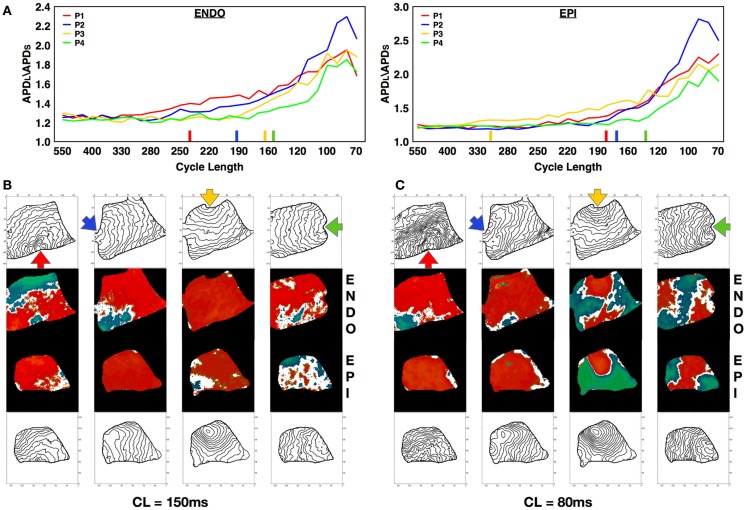
**Effect of pacing site on the development of alternans**. **(A)** APD dispersion amplitude for endocardium (main axis 7 cm) and epicardium (main axis 6 cm) from four different and orthogonal RV pacing sites: apex (P1, blue), posterior free wall (P2, green), base (P3, yellow), and anterior free wall (P4, red) (see Figure S1B in Supplementary Material for stimulus location). Colored bars on the x-axes indicate the points at which each curve has grown by more than 10% compared to its value at the longest CL. **(B,C)** Alternans maps and activation contour maps at CLs of **(B)** 150 ms and **(C)** 80 ms for endocardium and epicardium showing the differences obtained by pacing from the four distinct sites.

Alternans can be affected by intrinsic heterogeneities in the tissue, an effect that becomes apparent by altering the location of the stimulus (Pastore et al., 1999; Hayashi et al., 2007; de Diego et al., 2008; Ziv et al., 2009). To investigate how pacing site affects alternans dynamics in canine tissue, we mapped and measured alternans properties produced from pacing at four different RV locations: apex, posterior free wall, base, and anterior free wall in three preparations. We observed that varying the pacing site resulted in different onset CLs, amplitudes, and spatial patterns of alternans. Figure 7A shows a representative example of how the dispersion magnitude (difference between the maximum and minimum APD in the tissue) increased on the epicardium and endocardium as CL was decreased for the four different and orthogonal pacing sites: RV apex (P1, blue), RV posterior free wall (P2, green), base (P3, yellow), and RV anterior free wall (P4, red); see Figure S1B in Supplementary Material for anatomical identification. Figure 7A shows that alternans magnitude depended strongly on the stimulus location. Pacing from the posterior free wall produced the maximum alternans amplitude, whereas pacing from the opposite direction (anterior free wall) resulted in the smallest alternans amplitude. Pacing from the base and the apex gave similar intermediate amplitudes. These differences in alternans amplitude may have occurred because of differences in propagation arising from spatial variations in fiber orientation, which is more symmetric between base and apex compared to the anterior and posterior free walls. Figure 7A also shows that the rate of growth in the dispersion magnitude as CL was decreased changed depending on the pacing site and varied in this case by as much as 150 ms. For both the endocardium and the epicardium, the alternans magnitude grew more rapidly when pacing from the base than from any other location. Pacing from different sites also resulted in different patterns of alternans. Figures 7B,C demonstrate the differences in alternans patterns obtained by pacing from the four pacing sites for two different CLs (160 and 80 ms). Furthermore the activation isochrones in the contour plots show that fiber orientation along the RV tissue affected the pattern of activation and the conduction velocity depending on the pacing site and thus altered the patterns of discordant alternans. Further examples are shown in Figure S5 in Supplementary Material.

### 3.7 Effect of pacing history on alternans properties

**Figure 8:**
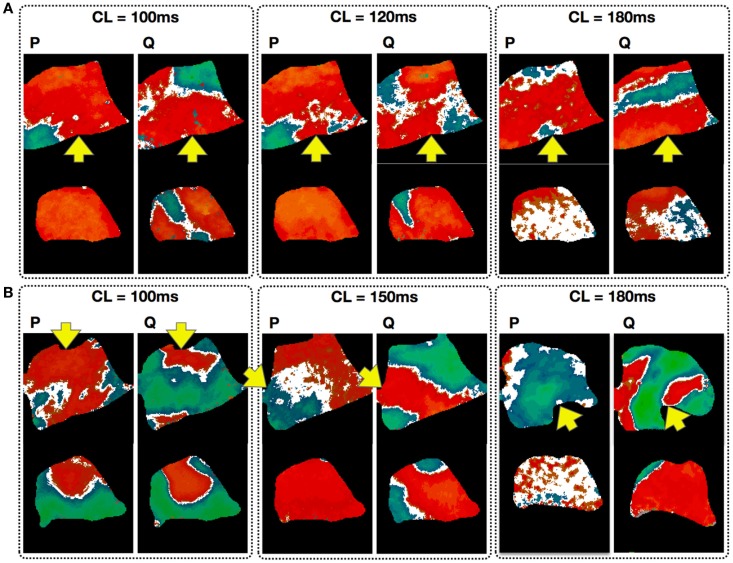
**Effect of pacing history on the development of alternans**. Each panel compares the endocardial (top; main axis 7 cm) and epicardial (bottom; main axis 6 cm) steady state patterns obtained via a pacedown (P-protocol) and a quiescence (Q protocol) with the same CL. Arrows indicate pacing site. **(A)** Alternans patterns in the same preparation for three CLs (100, 120, and 180 ms). **(B)** Maps taken from different preparations with the patterns resulting from different pacing sites for three CLs (100, 150, and 180 ms). Note that for the two cases with CL = 100 ms, the difference in pacing site affects the spatial pattern produced although the tissue preparation is the same.

Just as alternans properties such as CL onset for alternans and pattern morphology have a spatial dependency on the stimulation site, both pacing history and the dynamical state of the tissue before pacing can significantly affect how alternans develops (Mironov et al., 2008; Ziv et al., 2009). To quantify this phenomenon, we compared alternans maps obtained in the same tissue preparations (in 3 dogs, 524 recordings) for the same CL using two different protocols: a pacedown (P-protocol), where the tissue was paced initially at a CL of 550 ms with the CL slowly decreased (see methods) to the given CL, and by pacing at the given CL directly following quiescence (Q protocol). In both cases, measurements were performed at steady state. Figure 8 shows simultaneous steady state endocardial (top) and epicardial (bottom) recordings obtained using the P and Q protocols with the same CL. The top three panels show the differences in alternans patterns obtained in the same preparation for three CLs (100, 120, and 180 ms) applied to the same pacing site (base), whereas the bottom three panels show the patterns resulting from different pacing sites for three different CLs (100, 150, and 180 ms). Note that for the two cases where CL = 100 ms, the difference in pacing site affects the spatial pattern produced although the tissue preparation is the same. In nearly all cases more nodal lines appeared from the pacedown protocol (91% endo, 59% epi) than from the quiescent protocol (69% endo, 55% epi).

### 3.8 Initial conditions and multistability in cardiac tissue

**Figure 9:**
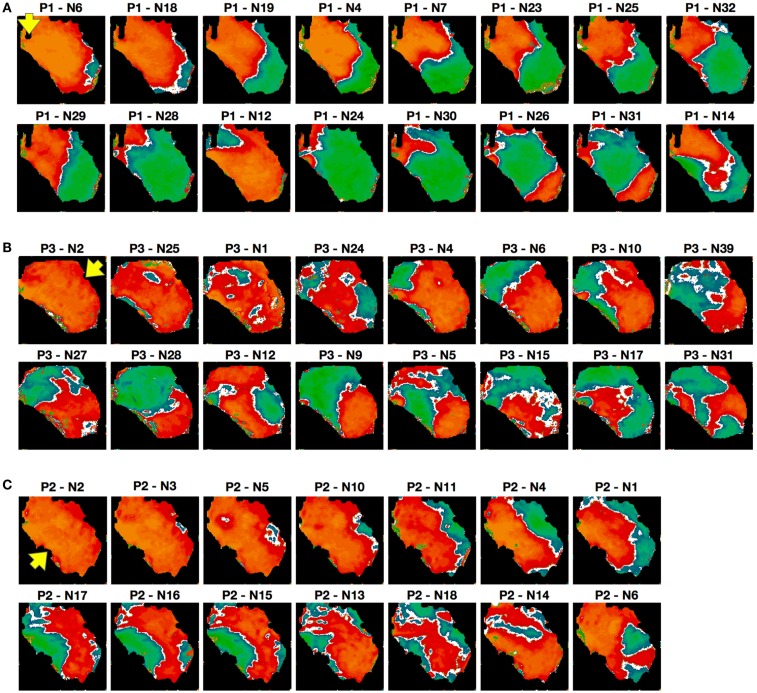
**Alternans patterns dependence on initial conditions**. Representative examples of alternans patterns obtained in a given preparation over a 20-min interval at a single CL of 150 ms; see text for details. To facilitate comparisons, the patterns are arranged in order of similarity with a main axis of 7 cm, but it is important to note that their appearance is not in chronological order (the experiment number is indicated on top of each panel). Pacing sites are indicated by the arrows [RV posterior in **(A)** apex in **(B)** and base in **(C)**].

Because alternans patterns differed depending on pacing site and stimulation protocol, we studied the sensitivity of alternans patterns to pacing history more generally. To analyze the effects of similar initial conditions on pattern formation, we paced 3 different RV preparations and obtained 126 recordings with similar initial conditions and constant CL. The Constant or (C protocol) consisted of pacing the tissue at a fixed CL for 20 s, followed by cessation of pacing for 20 s, during which time the tissue returned to quiescence, and then repetition of these two steps for 20 min, for a total of 30 repetitions. The first 10 s of each episode were recorded and the alternans maps corresponding to steady state (which was always reached within 10–15 beats; see Figure 1A; Figures S2D and S6 in the Supplementary Material) were constructed. We found significant variations in the alternans patterns over the different repetitions. Figure 9 shows selected examples of the diversity in patterns obtained with the constant protocol (CL = 150 ms) on the same preparation, with different pacing sites used in Figures 9A–C. Differences in the numbers and positions of nodal lines were observed, and even differences in the overall state of the tissue (concordant vs. discordant alternans) occurred. The pattern observed for a given repetition showed no obvious correlation with the patterns observed for the immediately preceding and following repetitions.

### 3.9 Pseudo-ECG signals during alternans

**Figure 10:**
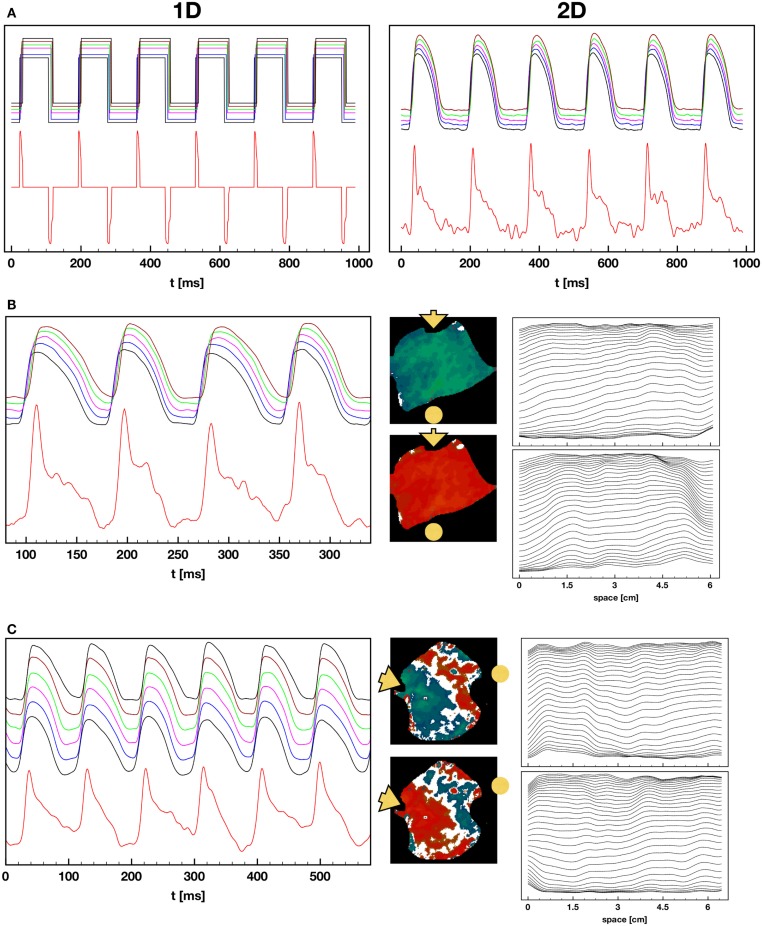

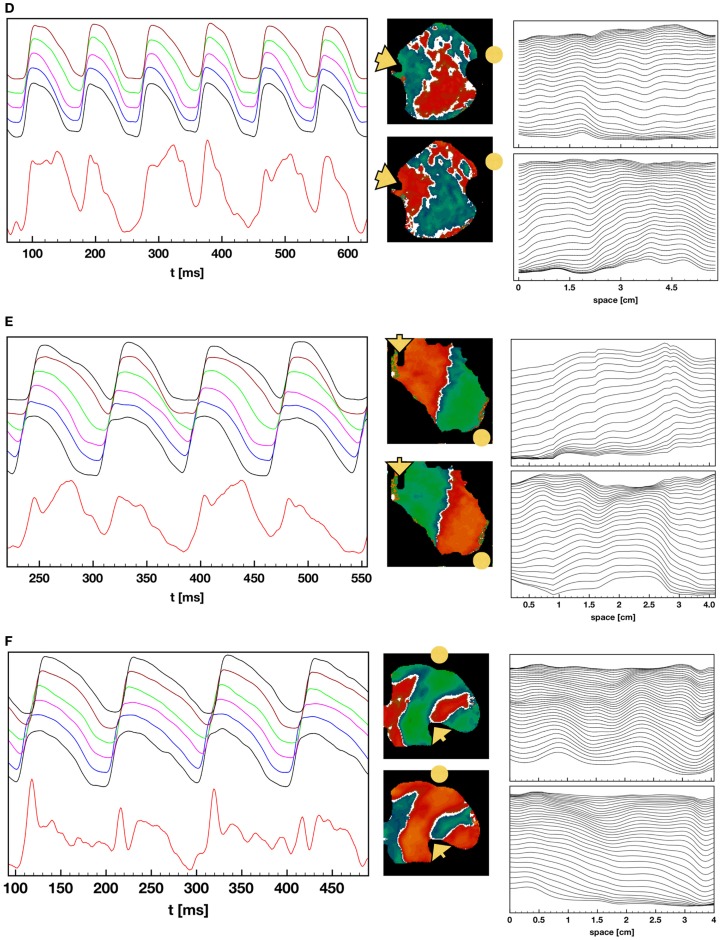
**Pseudo-ECGs during 1:1, 2:2 concordant, and 2:2 discordant AP propagation**. **(A)** Pseudo-ECG during normal 1:1 pacing. Left: pseudo-ECG (lower trace) obtained from a square wave representation of the APs (upper trace) obtained by converting the voltage to 0 for values below 25% repolarization and to 1 for values above that threshold. The binary signal minimizes noise effects and shows clear QRS complexes and inverted T waves, along with a clear return to baseline during the S-T segment. The upper plot shows APs obtained from several points in the tissue. Right: pseudo-ECG (lower trace) and optical action potentials (upper trace) from the original preparation. The pseudo-ECG is noisier, and features like the T waves are more difficult to determine. **(B)** Pseudo-ECG and AP plots as in **(A)** but during 2:2 concordant alternans. The center plot displays the difference in APDs in the tissue for two consecutive beats. The rightmost plot shows wave backs at multiple times along a line for the two consecutive beats, indicating that the tissue depolarizes and repolarizes in different ways for the two beats. Propagation is from left to right and the voltage values are separated 2 ms in time. **(C–E)** Pseudo-ECG, AP, APD difference, and wave back plots as in **(B)** but for three cases of discordant alternans, where the wave back dynamics is more complex between beats due to nodal lines. **(F)** Pseudo-ECG, AP, APD difference, and wave back plots as in **(B)** but for a particular case of discordant alternans, where multiple defined regions with opposite phase are present.

Pseudo-ECGs have been used to characterize the dynamics of propagating waves in simulated cardiac tissue (Aslanidi et al., 2005; Weiss et al., 2007; Bueno-Orovio et al., 2008). Although clinically the largest contribution to the ECG comes from the LV, a pseudo-ECG from an RV preparation provides useful information during concordant and discordant alternans. Figure 10 shows representative examples of calculated pseudo-ECGs (Clayton and Holden, 2002; Gima and Rudy, 2002) using RV preparations during 1:1, 2:2 concordant, and 2:2 discordant rhythms. Even during normal 1:1 propagation, the pseudo-ECGs obtained from RV preparations are different from standard ECGs in that they do not show a flat S-T segment, the T wave is inverted, and there is no clear separation between the S-T segment and the T wave.

To explain these differences, Figure 10A shows the pseudo-ECG calculated along a cable in the direction of propagation during a 1:1 rhythm. In the right panel, the action potential has been converted to a square wave: above a threshold of 25% repolarization, it is set to *V*_max_, and below that threshold, it is set to *V*_min_. In this case it is possible to obtain a clear QRS complex, flat S-T segment, and a clear T wave. The T wave inversion is expected because the tissue is relatively homogeneous electrophysiologically (Clayton and Holden, 2002; Bueno-Orovio et al., 2008). The right panel of Figure 10A shows the full pseudo-ECG from the same experiment shown in Figure 10A. Although there is a clear QRS complex, the S-T segment and T wave are merged, mostly because of the small amount of noise in the AP signal during the plateau and downstroke.

Figure 10B shows the pseudo-ECG during concordant alternans, which displays differences in the height and duration of the T wave during even and odd beats. Figures 10C–E show pseudo-ECGs for three different cases of concordant alternans. These pseudo-ECGs show not only alternating shapes between even and odd beats, as in the concordant alternans cases, but also more complicated morphologies and a failure to return to baseline during every other T-Q interval. This is because for the short cycle lengths at which DA appears, every two beats the wave that has a short APD at the end of the tissue has not left the tissue before the new wave has already started to propagate at the beginning of the tissue, so that the tissue is not completely polarized at any time during that cycle. During discordant alternans, the change in morphology of the AP during propagation along the tissue can produce a marked inflection point that can be present during the S-T segment, as shown in the plots of APs from different locations given on top of the pseudo-ECGs and the wave back plots in space shown to the right of the signals.

## 4 Discussion

Electrical alternans is a well known marker for electrical instabilities in cardiac tissue and has been amply correlated experimentally (Pastore et al., 1999; Choi et al., 2007; Ziv et al., 2009; Weinberg et al., 2010) and numerically (Karma, 1994; Qu et al., 2000; Watanabe et al., 2001; Fenton et al., 2002a; Cherry and Fenton, 2004) with the inducibility of arrhythmias. However, most studies of spatially extended alternans have been performed in monolayers and small mammalian hearts and often at lower temperatures where the alternans magnitude is larger. Only a small number of studies of alternans have been conducted in larger mammalian hearts such as dogs and pigs (Gilmore et al., 1967; Hashimoto and Nakashima, 1991; Murphy et al., 1996; Weiss et al., 2011), but in general those studies have been performed in the context of ischemia and without optical mapping, with the exceptions of a recent study on short-term memory in LV canine wedges (Zhang et al., 2011) and a study of equine atrial fibrillation (Fenton et al., 2008a), both using optical mapping. Therefore, very little is actually known about the spatiotemporal properties of alternans in ventricles of large mammals at physiological temperatures. In this study, we have presented results from 1362 different experiments involving dual mapping of the epicardium and endocardium from 9 canine right ventricular preparations. As in monolayers (Gilmore et al., 1967; Bien et al., 2006; Weinberg et al., 2010), smaller hearts (Pastore et al., 1999; Walker et al., 2003; Choi et al., 2007; Mironov et al., 2008; Ziv et al., 2009), and numerical studies (Qu et al., 2000; Watanabe et al., 2001), alternans in RV preparations also develops as the pacing cycle length decreases, where concordant (Figure 2B) and discordant alternans (Figure 2C) patterns are present and can lead to fibrillation (Figure 1B). However the range of CLs for which alternans is present is much larger for canine RV (about 300 ms; with 200 ms for discordant alternans) compared for example with rabbit ventricles [75 ms (Mironov et al., 2008; Zhao, 2008); with 10–50 ms for discordant alternans (Hayashi et al., 2007; Mironov et al., 2008; Zhao, 2008)] and transgenic rabbits with LQT2 (about 110 ms; with 60 ms for discordant alternans) (Mironov et al., 2008). In addition, we found that the alternans onset in RV preparations appeared first on endocardial tissue at larger cycle lengths compared to epicardium, in agreement with previous results on epicardium and endocardium single cell studies (Cordeiro et al., 2007) and in canine ventricles under long QT conditions (Shimizu and Antzelevitch, 1999), and compared to smaller mammalian hearts a much richer and more complex scenarios emerge even at normal physiological temperatures as we discuss below.

### 4.1 Nodal line dynamics

The properties of nodal lines have been studied previously and recently their dynamics have been used to try to classify the underlying alternans mechanism (Hayashi et al., 2007; Mironov et al., 2008; Zhao, 2008). The first optical mapping experiments describing alternans (Pastore et al., 1999) considered anatomical tissue heterogeneity as the mechanism underlying discordant alternans. Computational and theoretical studies subsequently showed how variations in conduction velocity (CV) could account for the transition from concordant to discordant alternans, without the need for intrinsic heterogeneities (Qu et al., 2000; Fox et al., 2002; Ramshesh and Knisley, 2006). Hayashi et al. (2007) recently suggested, based on a computational study, that the difference between these two possible mechanisms could be distinguished by analyzing the stability of the nodal lines. In agreement with previous computational studies, they showed that when discordant alternans was formed by the CV mechanism, nodal lines moved toward the pacing site as the pacing rate was increased, regardless of whether the alternans arose from a voltage (steep APD restitution) or calcium (steep function governing Ca^2+^ release from the SR) mechanism. Their study also showed that when alternans was due to tissue heterogeneities, nodal lines behaved differently. Specifically, when DA arose from heterogeneities in calcium the nodal lines were stable and did not move even when the CL was changed, whereas when DA arose from heterogeneity in APD restitution, the nodal lines were unstable and drifted away from the pacing site. Based on these results, Hayashi et al. (2007) suggested that nodal line dynamics could be unequivocally used to classify the mechanism underlying alternans in a particular preparation. They concluded, using their criterion, that in rabbit ventricles, CV restitution was the mechanism underlying DA, because nodal lines migrated toward the pacing site as the CL was decreased.

Ziv et al. (2009) and Mironov et al. (2008) reached a similar conclusion that CV restitution-induced alternans caused nodal lines to move toward the pacing location. However, Mironov et al. (2008) also found that in some cases nodal lines in rabbit ventricles could remain stable after decreasing the pacing CL or could drift in different directions, either toward or away from the pacing site. Because nodal lines did not always move toward the pacing site, and because in the regions featuring this behavior it took longer for the APD to accommodate to changes in CL, they concluded that the unstable behavior of the nodal lines stemmed from adaptation and short-term memory. However, no mechanism for the nodal line behavior was proposed. Similarly, Ziv et al. (2009), observed movement of nodal lines in transgenic rabbit ventricles, and although short-term memory is considered to operate on a time scale from seconds to minutes, they concluded that because nodal line shifts took place 10–30 s after the change in CL, they most likely were due instead to the larger electrophysiological heterogeneities of the LQT2 hearts.

Although identification of mechanisms as a function of nodal line behavior would be of extreme value, the markers used so far have serious limitations. For example, nodal lines produced by CV restitution do not need to be static with a fixed CL, as there are cases were they actually move. The simplest example of this is alternans on a ring, where unless the ring length matches the alternans amplitude, the node will move (which is usually the case). Although in this case movement of nodal lines is an effect of the periodic boundary conditions, Watanabe et al., 1995 nodes can move even in open 1D cables (or 2D sheets or 3D slabs) (Watanabe et al., 1995) because of electrotonic currents. Non-static nodes have been shown numerically (Fenton et al., 2008b) and experimentally in Purkinje fibers (Christini et al., 2006) (where nodes migrate to the stimulation site, disappear, re-form again far from the pacing site, and repeat the migration). Furthermore, nodes can also remain stationary and not move toward the pacing site after changes in CL when small heterogeneities are present that can pin the nodes (Watanabe et al., 2001). Another difficulty in applying the approach of Hayashi et al. (2007) for classifying alternans mechanisms is that their simulations with heterogeneity considered only linear gradients in electrophysiological properties in one direction. Different gradients, including non-linear gradients (which are often present) (Glukhov et al., 2010), anisotropic fiber direction and rotation (Fenton and Karma, 1998), and effects of three-dimensionality were not considered. All of these factors could have additional effects on the behavior of nodal lines. Taken together with the observations of multiple types of nodal line behavior for example in rabbit ventricles (Hayashi et al., 2007; Ziv et al., 2009), it seems unlikely that a particular mechanism for alternans can be identified solely by observing the nodal line dynamics. Instead, other information, such as electrotonic currents (Cherry and Fenton, 2004; Fenton et al., 2009), memory (Cherry and Fenton, 2004; Kalb et al., 2004), and detailed 3D structural and heterogeneity distributions (Glukhov et al., 2010), may in fact be needed to differentiate among alternans mechanisms.

In our experiments, we did not observe traveling nodes. Rather, nodal lines formed relatively quickly after a few beats at a given CL and remained stationary for a constant CL (see, for example, Figures S2 and S6 in Supplementary Material). As the CL was decreased, we occasionally observed migration of the nodes in the direction of the pacing site, as shown in Figure 5A. However, in most cases the nodal lines were predominantly stationary, and as the CL was decreased further, more complex nodal structures appeared, including islands and additional lines (see Figures 4A,B). Stable nodes were observed 57% of the time on the endocardium with 7% showing nodal lines moving away from the pacing site and 36% toward the pacing site, while for the epicardium 78% of the nodes were stable with 7% moving away from the pacing site and 15% toward the pacing site. The presence of extra nodal lines allowed for the creation of multiple discordant alternans regions (Figures 2D, 4B, 5A, 6B, and 7B) and very complex patterns (Figures 8 and 9). Although some of the studies in rabbit ventricles showed that multiple nodal lines could appear (Hayashi et al., 2007; Mironov et al., 2008; Ziv et al., 2009), they were transient, as they drifted to a boundary and disappeared, so it is possible that the combination of the large canine RV size and wavelength allowed the formation of these configurations with multiple stable nodal lines, as in some monolayer experiments (Christini et al., 2006).

Although numerical and some experimental studies have shown that nodal lines are formed perpendicular to the direction of the pacing site (as shown in Figure 2C), we found that this was not always the case. Nodal lines could appear in any direction with relation to the pacing site, as shown in Figures 2B and 4A (CL 210–190 ms), and Figures S7A,B in Supplementary Material, among others. The study of Ziv et al. (2009) with transgenic LQT rabbits reported nodal lines that were not perpendicular to the pacing site and they attributed the orientation of nodal lines to the heterogeneity of the tissue arising from LQT2 and not to CV restitution. Specifically, they postulated that if CV restitution drives the onset of DA, the two regions of DA would be separated by slow conduction and therefore DA nodal lines would align with activation isochronal lines. We believe that this may not be completely accurate, as the only requirement is for the CV at the nodal line not to be slow or fast but to be similar at each beat and for the CV magnitude to alternate on opposite sides. Once three-dimensionality, fiber direction and rotation, and heterogeneities are accounted for, nodal lines may not need to align to isochronal activations at a given surface. Although it is difficult to indicate exactly which mechanism(s) drives alternans in canine RV preparations and our temporal resolution (2 ms) is not always sufficient to obtain precise CV curves, in many cases we can calculate alternans CV plots that clearly show induction by the mechanism of CV restitution (see Figure S8 in Supplementary Material).

### 4.2 3D Nodal surfaces

By simultaneously recording from the epicardium and endocardium, we were able to correlate the discordant alternans patterns between the two surfaces. Although in some cases the nodal lines on the surface almost certainly were associated with nodal surfaces in three dimensions that were straightforward extensions through the ventricular wall (similar to I-shaped filaments of scroll waves in 3D), as seen in Figure 4A (CL 140 ms) and Figure 7C (P4), in most cases there was a substantial difference between the patterns on the epicardial and endocardial surfaces (see Figures 4A,B and 8, among others). Thus, it is expected that the nodal surfaces can have a very complicated intramural structure, just like scroll waves (Biktashev et al., 1994; Fenton and Karma, 1998; Cherry and Fenton, 2008), where they can bend, fold, and touch both surfaces, or terminate at one of the sides normal to the epicardium or endocardium of the ventricular section, or attach to only one surface to form concave or convex nodal surfaces (similar to U-shaped filaments of scroll waves). These dynamics probably are produced by the transmural heterogeneity in APD dispersion along the thickness of the ventricle as well as by fiber rotation. We showed differences in nodal line patterns on the epicardium and endocardium (see Figure 3), which most likely arise from differences in AP dynamics due to different expressions of ion channels and Ca^2+^ transients on the epicardium, endocardium, and mid-myocardium Laurita et al. (2003) and Cordeiro et al. (2004, 2007). Such differences could, in turn, lead to differences in the CLs between layers for which alternans can be supported, which would affect the topology of nodal lines as they develop. For example, if for a given CL the endocardium can sustain discordant alternans and the epicardium can sustain only concordant alternans, then the nodal surface produced at the endocardium topologically would be restricted either to form a concave island at the surface or to bend and terminate at one of the perpendicular edges. Numerical, intramural (Allison et al., 2007; Glukhov et al., 2010), and transillumination (Baxter et al., 2001; Hwang et al., 2006; Ramshesh and Knisley, 2006; Bernus et al., 2007; Mitrea et al., 2009) studies are needed to fully characterize the dynamics and effects of nodal surfaces on arrhythmia initiation.

### 4.3 Period-doubling route to chaos for VF?

Although multiple routes to chaos exist, the most commonly observed route in dynamical systems is a period-doubling cascade (May, 1976; Schuster, 1995; Shiferaw et al., 2005). For cardiac tissue, however, the type of bifurcation that leads to complex spatiotemporal dynamics during fibrillation still has not been well characterized, not only because multiple mechanisms can lead to fibrillation (Fenton et al., 2002a; Ideker and Rogers, 2006; Nash et al., 2006), but because different bifurcation routes have been implicated in the transition from period 1 to 2 (alternans), to higher-order rhythms and to chaos (Hastings et al., 2000) even under the same condition of periodic rapid pacing. The first transition (1:1–2:2) as the pacing period is decreased can be modeled by a simple one dimensional iterative map of the action potential duration (Nolasco and Dahlen, 1968; Fenton et al., 2002b) or intracellular calcium (Eisner et al., 2000; Qu et al., 2007) and was originally described experimentally and numerically as a supercritical period-doubling bifurcation (Guevara et al., 1984; Chialvo et al., 1990; Lewis and Guevara, 1990; Hall et al., 1999). However, later studies suggested a subcritical Hopf bifurcation (Gottwald, 2008) and intermittency (Shiferaw et al., 2005), which can develop when considering other effects such as memory, spatial coupling and calcium dynamics in the analysis. Similarly, the transition from 2:2 to higher-order rhythms could have multiple sources. Transitions to 2:1, 3:2, and other Wenckebach rhythms, which are not typical of many other dynamical systems, are present in cardiac dynamics as a result of the discontinuity in the response due to refractoriness and conduction blocks (Lewis and Guevara, 1990; Bien et al., 2006). In space, these *m:n* transitions, where *m* > *n* far from the pacing site and *m* = *n* at or near the pacing site, can lead to the development of alternans that is different from the alternans described in this manuscript. In those cases a pseudo-alternans is created at the border between the *m* = *n* and *m* > *n* regions, which we refer to here as Gaskell alternans, as he was the first to suggest that mechanical alternans could be the result of a 2:1 sub-region in an otherwise 1:1 domain (Gaskell, 1882). Gaskell-type alternans of APD was first described numerically by Arce et al. (2000) and then experimentally by Fenton et al. (2008a) in equine atria and later by Myles et al. (2008) in rabbit ventricles with myocardial infarction. It is possible that Gaskell alternans is the type of alternans most commonly observed during ischemia. Although 2:2 rhythms most commonly are followed by Wenckebach rhythms (Hescheler and Speicher, 1989; Arce et al., 2000, 2007; Bien et al., 2006), higher-order rhythms of 4:4 and 8:8 (see Figure 6) have been observed in other cardiac preparations during fast pacing, such as in bullfrog ventricles at room temperature (Savino et al., 1989), strips of sheep fetal epicardial ventricular tissue (Biktashev et al., 1994), and up to 16:16 rhythms in canine Purkinje fibers (Gilmour et al., 1997). In these cases, while most of the tissue may follow a period-two response, some regions can develop additional spatial bifurcations of the local rhythm and develop into higher-order period-doubling rhythms. We believe that the 4:4 and 8:8 rhythms presented here (Figure 6; Figures S3 and S4 in Supplementary Material) are the first higher-order rhythms obtained in large perfused sections of mammalian ventricles at physiological temperatures. Because the range of CLs over which a given period-doubling bifurcation can be observed decreases exponentially with increasing periodicity, it is not surprising that higher-order rhythms (4:4, 8:8, and 16:16) are not often recorded; in our case we detected higher-orders in only 2% of DA episodes. Nevertheless, the fact that they have been observed suggests that in some cases the transition to fibrillation (particularly during ectopic beats) may be the result of a series of period-doubling bifurcations (see Figure S10 in Supplementary Material). However, because other higher-order rhythms such as 3:3, 6:6, and 3:1 also have been observed, it is possible that other attractors can co-exist (Savino et al., 1989; Lewis and Guevara, 1990), as well as complex dynamics caused by biphasic APD restitution (Watanabe et al., 1995; Qu et al., 1997; Fenton et al., 2002a) or supernormal CV restitution (Fenton et al., 2002a; de Lange and Kucera, 2010; Echebarria et al., 2011; Kim et al., 2013).

### 4.4 Alternans patterns as a function of spatial heterogeneity and initial conditions

Although a number of studies have characterized alternans development as a function of pacing CL, little is known about the effect of initial conditions or spatial heterogeneity on alternans dynamics. Pastore et al. (1999) observed that the discordant alternans pattern in guinea pig ventricles was consistently oriented along the apex-to-base axes regardless of the pacing site and attributed this to intrinsic electrophysiological heterogeneity. In contrast, Hayashi et al. (2007) observed a change in the position of the nodal line in rabbit ventricles when the stimulation site was changed. Explanations for the difference between these two studies include the endocardial cryoablation of the guinea pig experiments, which may have imposed a static heterogeneity, and differences in species and tissue size, as the dynamics of alternans in smaller tissues is more restricted. Given that the space constant in cardiac tissue is about 1 cm, the smoothing effects of electrotonic coupling on APD dispersion are more pronounced in smaller hearts. More recently, however, Ziv et al. (2009) showed that pacing from different sites in normal rabbit ventricles also changed the orientation of nodal lines, in agreement with Hayashi et al. (2007), but the orientation did not change in ventricles obtained from transgenic rabbits with LQT2. In the latter, the mechanism of discordant alternans and consistency of nodal line patterns was attributed to tissue heterogeneity, rather than conduction velocity restitution.

In the present study, we also observed differences in nodal line dynamics depending on the stimulation site, which may be explained by larger tissue size, where not only are electrotonic effects reduced, but anisotropy and fiber direction extend over a large area and can modify wave propagation depending on the orientation of the pacing site with respect to local fiber direction (see Figure 7), thereby supporting more complicated patterns. Along with pacing site location, we found that temporal initial conditions had a strong effect on alternans inducibility and nodal line patterns. Our results are similar to those of Ziv et al. (2009), who showed that in normal rabbit ventricles a pacedown protocol in which the CL was decreased by 10 ms did not produce discordant alternans as often as a protocol in which the CL was decreased by 2–5 ms. We found that initial conditions corresponding to a pacedown and to quiescence usually resulted in very different patterns of alternans and, in some cases, in the development of concordant rather than discordant alternans (see Figure 8). Although it could be argued that the difference obtained between the two protocols was due to short-term memory, we found that the same quiescent protocol applied to the same preparation produced a different pattern of alternans each time it was applied (Figure 9). These results imply that the development of alternans patterns is very sensitive to small perturbations in the initial state of spatial dispersion within the tissue. In one of the first studies of alternans with optical mapping, Laurita et al. (1996) showed that a single premature beat could influence the dispersion of refractoriness by enhancing repolarization gradients along the tissue and that the enhanced dispersion was very sensitive to the timing of the premature stimulus. Similarly, one of us showed numerically in Watanabe et al. (2001) that a premature beat could change the repolarization properties of the tissue enough to initiate discordant alternans after one beat. It follows that the repolarization state of the tissue could have a significant effect on the type of alternans (CA or DA) and on the spatial pattern, and that, as in chaotic systems, a very small perturbation of the initial conditions could lead to very different patterns and dynamics. These dynamics could be attributed to the different space constants for voltage and calcium. While voltage is coupled in space with a space constant on the order of millimeters, calcium can vary substantially locally even within a myocyte (Gaeta et al., 2009; Tsai et al., 2011) and variations in calcium concentration in space have been shown numerically to lead to different distributions of alternans, even for the same protocol (Zhao, 2008). Therefore, this effect needs to be studied in more detail both computationally and experimentally with simultaneous voltage and calcium recordings.

Overall, we have found that spatial alternans properties in canine RV preparations are very sensitive to initial conditions and pacing site, given that they depend on small variations in intrinsic repolarization properties and the direction of propagation. This sensitive dependence on initial conditions may be the cause for the extended transition of concordant alternans reappearing following discordant alternans, as for a given CL multiple solutions can exist (Figure 9; Figure S9 in Supplementary Material) and small variations during the change of CL can lead to a different solution and the alternans can change from a discordant pattern to a concordant one (see, for example, Figure 9B, N2 and N6 and Figure 9C, N2 and N4), small variations therefore would change a normal transition to an extended one (Figure 5).

### 4.5 Limitations

In this study only right ventricles were used, and thus our results may not extend to the left ventricle or to the whole heart. Pacing was always performed on the endocardium and never on the epicardium; therefore, the fact that alternans generally developed sooner on the endocardium may only be an effect of the pacing site. However, because the RV is relatively thin and contour lines on the epicardium showed that the activation quickly reached the other surface essentially as a point stimulus, we believe that our results would not change appreciably were the epicardium paced instead of the endocardium. The intracellular calcium concentration was not optically recorded in this study, but this information would help to clarify the roles of voltage and intracellular calcium in alternans development and also could help to elucidate how islands and nodal surfaces form (because voltage and Ca^2+^ have different space constants, it is possible that they could be decoupled). Finally, our results were obtained in one type of canine preparation, as only beagle hearts were used in this study.

## 5 Conclusion

Through analysis of more than 1300 optical mapping recordings of 5–10 s each in canine RV preparations, we have shown the following (a comprehensive summary of the quantitative results is shown is Table 1). (i) Both concordant and discordant alternans are readily observed at physiological temperature. (ii) As the pacing cycle length is decreased, alternans (2:2 rhythm) predominantly develops sooner on the endocardium than on the epicardium. (iii) The transition from normal rhythm to concordant alternans to discordant alternans and finally to fibrillation or conduction block as CL is decreased may be extended by the appearance of second regions of concordant and discordant alternans. (iv) Multiple stationary nodal lines can exist and need not be perpendicular to the pacing site or to each other. (v) Higher-order rhythms such as 4:4 and 8:8 occur in these large tissue sections from mammalian hearts at normal body temperature, an observation that suggests that in some cases the transition to fibrillation may arise from a series of period-doubling bifurcations. (vi) Discordant alternans has different dynamics for the epicardium and endocardium, leading to nodal surfaces with complex three-dimensional dynamics that can give rise to transmural and intramural, convex, or concave shapes that attach to only one surface forming islands. (vii) The complex spatiotemporal patterns observed during alternans are a function of both the site of stimulation and the stimulation history. Based on these results, the prediction of alternans pattern formation may not be a trivial task because of the high sensitivity to initial conditions, which may indicate some underlying predisposition to chaos. Therefore the answer to questions such as those posed by Zipes (2003) as to why some PVCs induce VT/VF but others do not, or why a patient died on Tuesday and not on Monday or Wednesday, may depend strongly on the precise state of the heart once an arrhythmogenic substrate has developed. Given that predicting the precise evolution of spatiotemporal dynamics may not be possible, as suggested by our results, efforts that focus on avoiding the creation of an arrhythmogenic substrate or on identifying interventions that render these substrates less sensitive to initial conditions may be more successful as antiarrhythmic approaches.

**Table 1:** **Summary of results**.

	ENDO	EPI
Number of recordings	681	681
Alternans development somewhere in the tissue at CL of 400 ms	65%	25%
Alternans development somewhere in the tissue at CL of 300 ms	100%	21%
Alternans development somewhere in the tissue at CL of 200 ms	100%	100%
Recordings showing full CA over the entire tissue before DA developed	78%	50%
Extended transition cases (where CA → DA → CA → DA) as CL decreased	21%	14%
DA cases where the nodal lines were perpendicular between epi and endo	8%	–
DA cases with more than 1 nodal line	79%	57%
Stable nodal lines (nodal lines that did not move at a given CL)	57%	78%
Moving nodal lines (toward the pacing site)	36%	15%
Moving nodal lines (away from the pacing site)	7%	7%
Development of more than one nodal line during DA (pace down protocol)	91%	59%
Development of more than one nodal line during DA (quiescent protocol)	69%	55%

## Conflict of Interest Statement

The authors declare that the research was conducted in the absence of any commercial or financial relationships that could be construed as a potential conflict of interest.

## Supplementary Material

The Supplementary Material for this article can be found online at: http://www.frontiersin.org/Cardiac_Electrophysiology_/10.3389/fphys.2013.00071/abstract
