# Supplementary material for: Effects of Pacing Site and Stimulation History on Alternans Dynamics and the Development of Complex Spatiotemporal Patterns in Cardiac Tissue
Source: Front Physiol. 2013 Apr 19;4:71. doi: 10.3389/fphys.2013.00071 (PMC3630331; doi:10.3389/fphys.2013.00071)
Supplement: Supplementary file 1 [file 41794_Fenton_Presentation1.PDF]

Effects of pacing site and stimulation history on alternans dynamics and the development of complex spatiotemporal patterns in cardiac tissue

A. GIZZI[1,2], E.M. CHERRY[3,4], R.F. GILMOUR, JR.[4], S. LUTHER[4,5], S. FILIPPI[1,6], AND F.H. FENTON[5,7] ∗,

[1]Nonlinear Physics and Mathematical Modeling Lab, University Campus Bio-Medico of Rome, I-00128 Rome, Italy
[2]Alberto Sordi Foundation, Research Institute for Aging, I-00128, Rome, Italy
[3]School of Mathematical Sciences, Rochester Institute of Technology, Rochester, New York 14623, USA
[4]Department of Biomedical Sciences, Cornell University, Ithaca, New York 14853, USA
[5]Max Planck Institute for Dynamics and Self-Organization, Am Fassberg 17, D-37077 Gttingen, Germany
[6]I.C.R.A. University of Rome "La Sapienza", I-00185 Rome, Italy
[7]School of Physics, Georgia Institute of technology, Atlanta, GA 30332, USA

Supplemental Material

∗Corresponding author. Email: flavio.fenton@physics.gatech.edu

© Harvard Science.

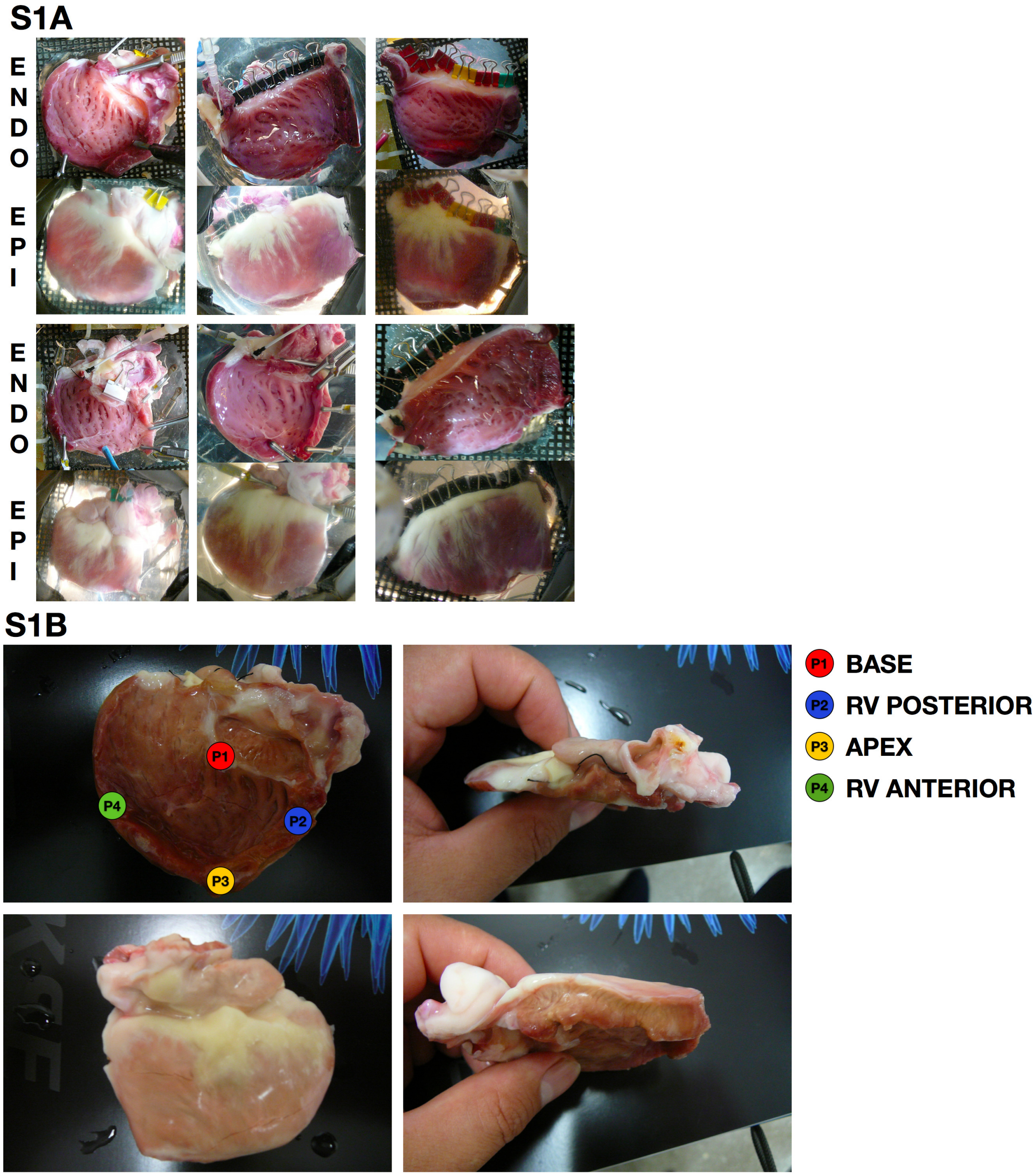

FIG. 1. **S1. Tissue preparations.** A) Endocardial and epicardial views of six of the nine canine right ventricular preparations adopted for optical mapping analysis. B) Frozen RV tissue with identification of the four pacing positions adopted for tissue stimulation: P1 base (red), P2 RV posterior (blue), P3 apex (yellow), and P4 RV anterior (green). The main axis of the optically mapped surface is 7 cm for the endocardium and 6 cm for the epicardium.

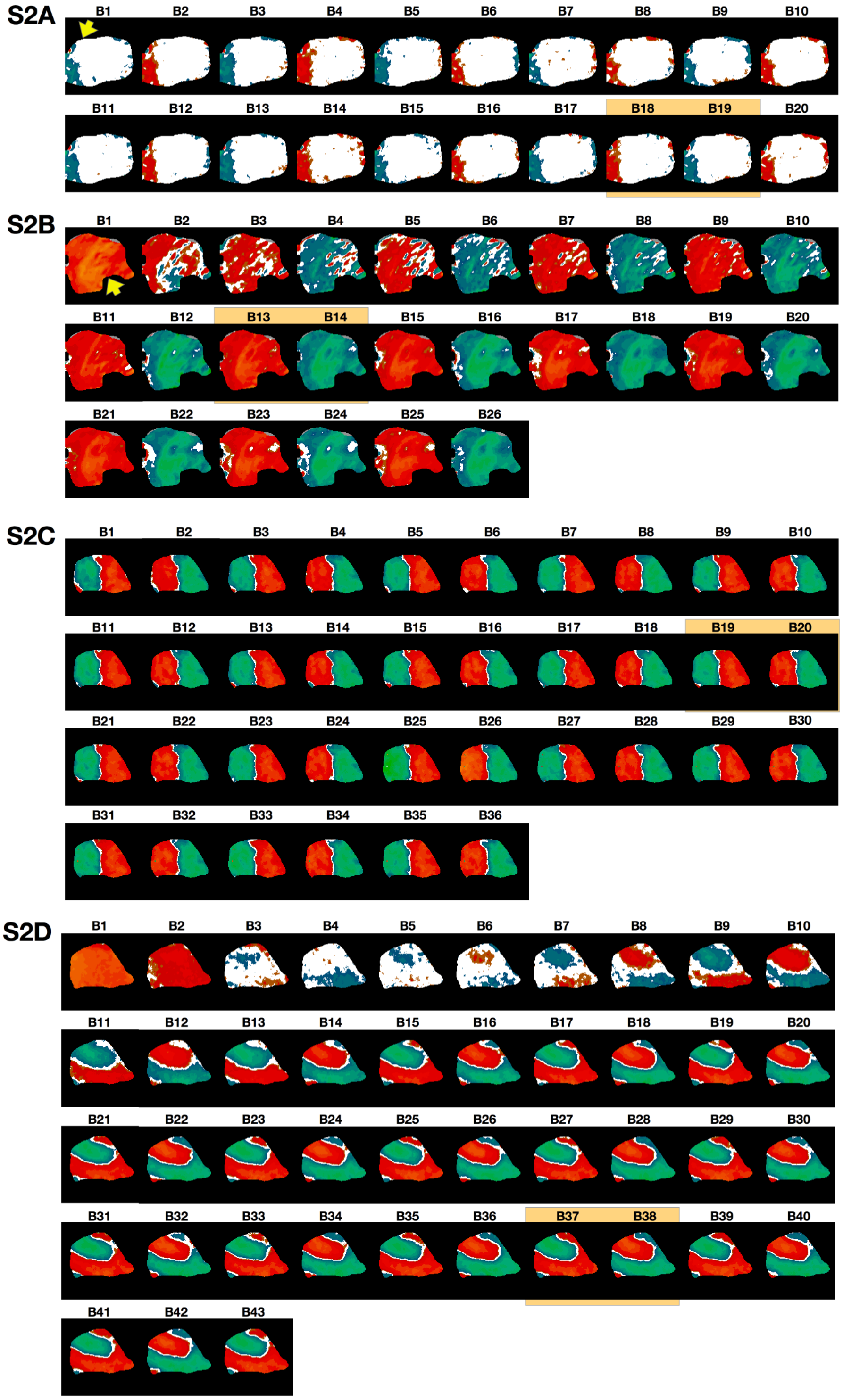

FIG. 2. **S2. Steady-state alternans patterns.** Complete sequence of spatial alternans maps shown that the steady-state is reached after few beats. Yellow arrows indicate pacing site. A) Endocardial patterns in the case of alternans concordant alternans onset at $CL = 400\,ms$ on a portion of the tissue; beats 18 and 19 are selected for Fig. 2A in the text. B) Endocardial patterns in the case of full concordant alternans at $CL = 220\,ms$; beats 13 and 14 are selected for Fig. 2B. C) Epicardial patterns of discordant alternans with one straight nodal line at $CL = 110\,ms$; beats 19 and 20 are selected for Fig. 2C. D) Epicardial patterns of discordant alternans with multiple nodal lines at $CL = 150\,ms$; beats 37 and 38 are selected for Fig. 2D.

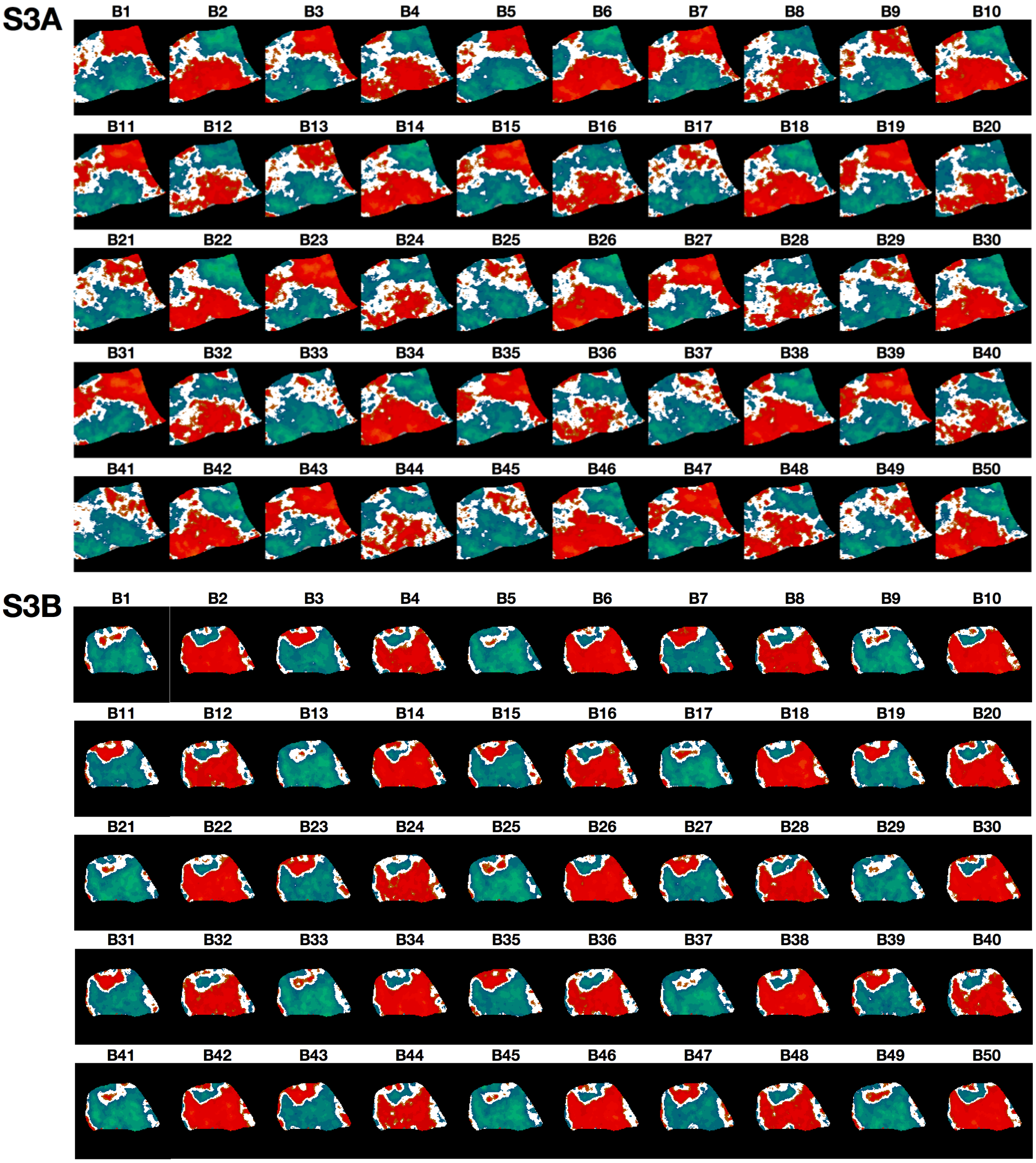

FIG. 3. **S3. Higher order rhythms in spatial patterns.** Complete sequence of alternans maps showing higher-order space-time dynamics. A) Endocardial maps at CL $= 100ms$ with a $4:4$ rhythm. B) Epicardial maps at CL $= 70ms$ with mixed regions of $2:2$ and $4:4$ rhythms.

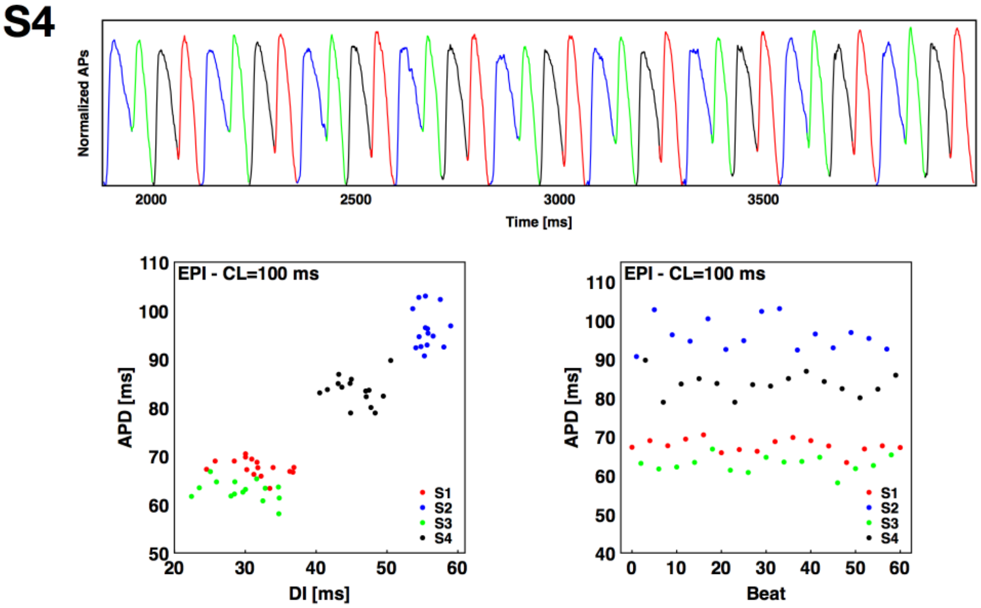

FIG. 4. **S4. Higher order rhythms in time.** Temporal distribution of the 4:4 degenerate case obtained at CL $= 100\,ms$ and discussed in Fig. 6C. The top panel highlights the four different action potentials by four different colors. The bottom panels show the APD vs. DI (left) and APD vs. beat number (right) distributions, confirming that the observed rhythm was a 4 : 4 and not a 3 : 3.

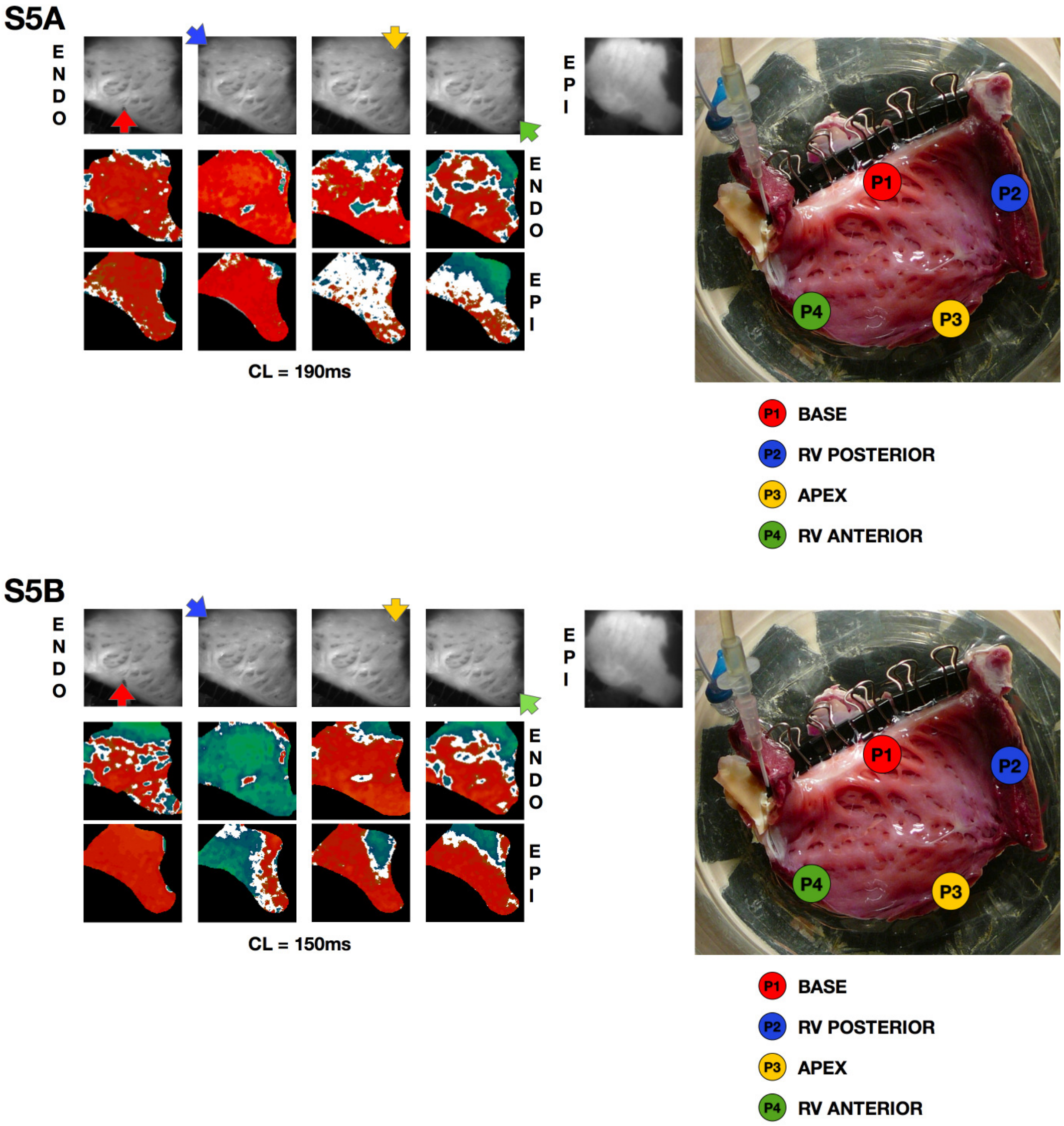
S5A
ENDO
EPI
ENDO
EPI
CL = 190ms
P1
P2
P3
P4
P1 BASE
P2 RV POSTERIOR
P3 APEX
P4 RV ANTERIOR
S5B
ENDO
EPI
ENDO
EPI
CL = 150ms
P1
P2
P3
P4
P1 BASE
P2 RV POSTERIOR
P3 APEX
P4 RV ANTERIOR

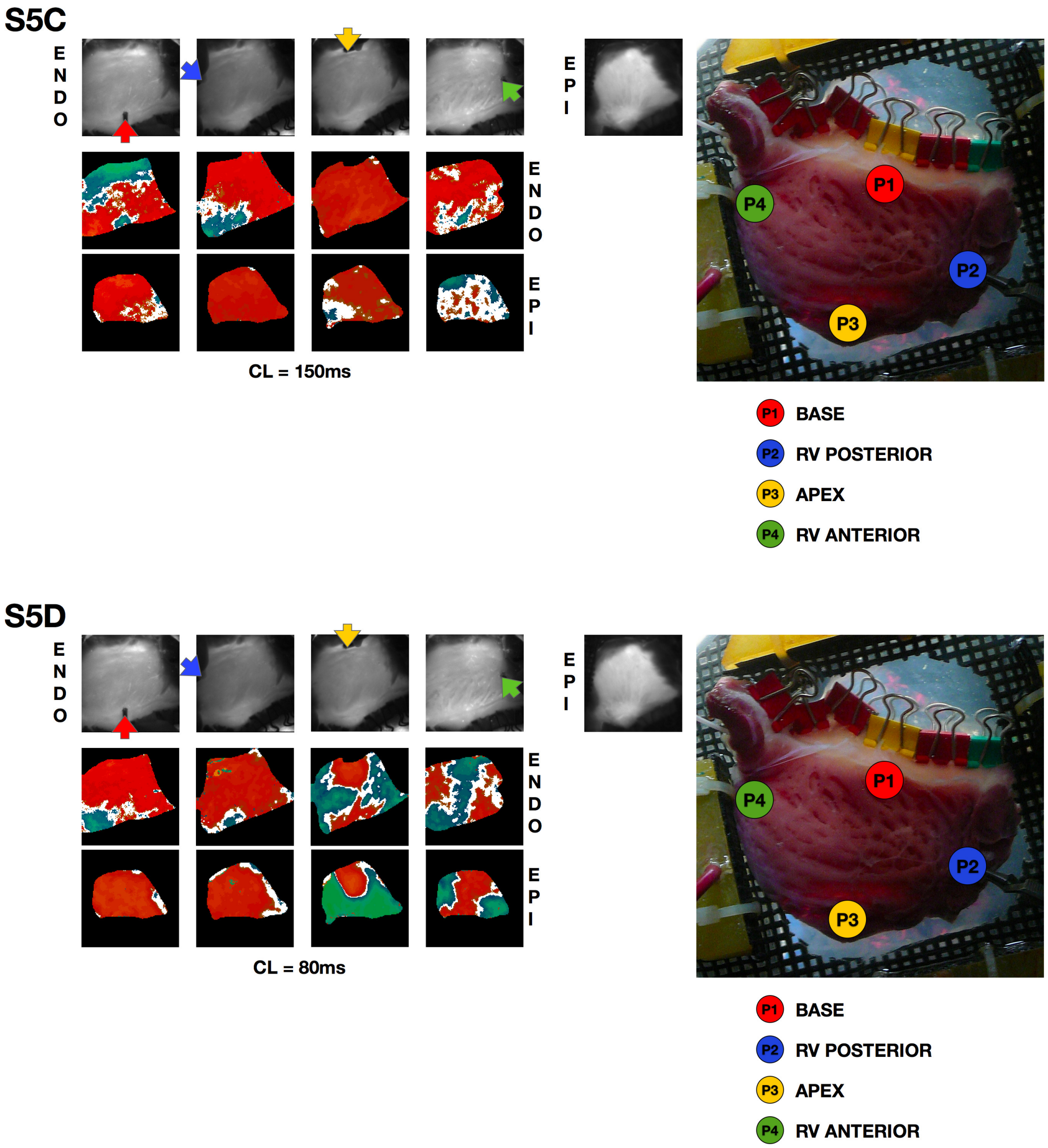

FIG. 5. **S5. Alternans patterns dependence on stimulation site.** Endocardial and corresponding epicardial maps depending on pacing site for two different tissue preparations. A- B) Same RV preparation paced at CL = 190 and 150 ms. C-D) Same RV preparations paced at CL = 150 and 80 ms.

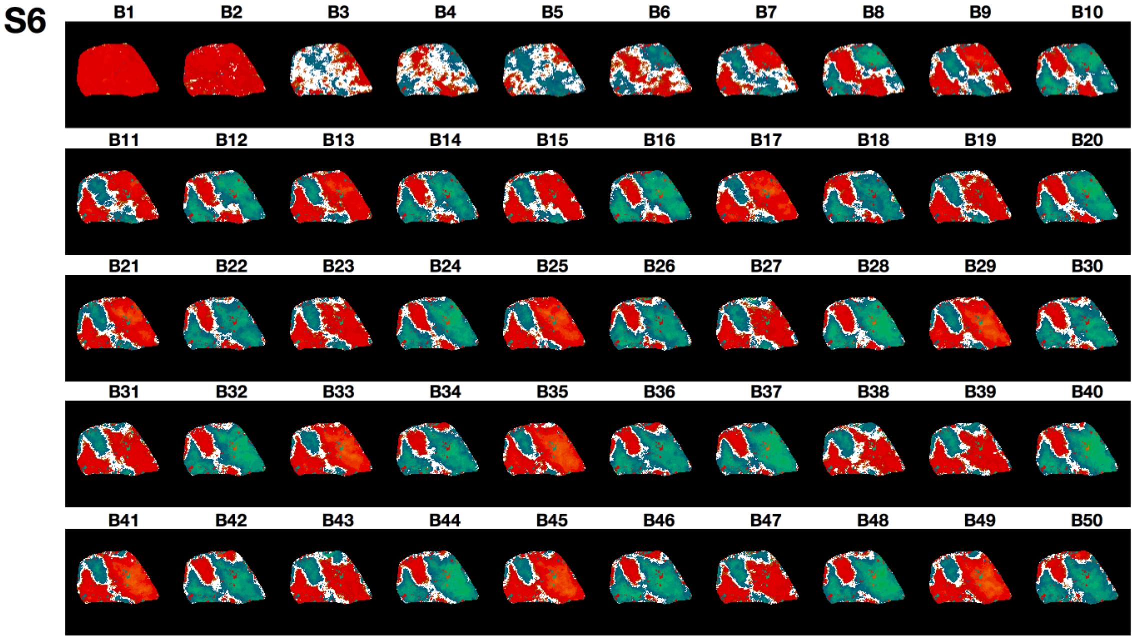

FIG. 6. **S6. Stationary nodal lines.** Complete sequence of epicardial alternans patterns obtained at $\mathrm{CL} = 100\,ms$ showing that the steady-state patterns form relatively quickly and do not move towards the pacing site.

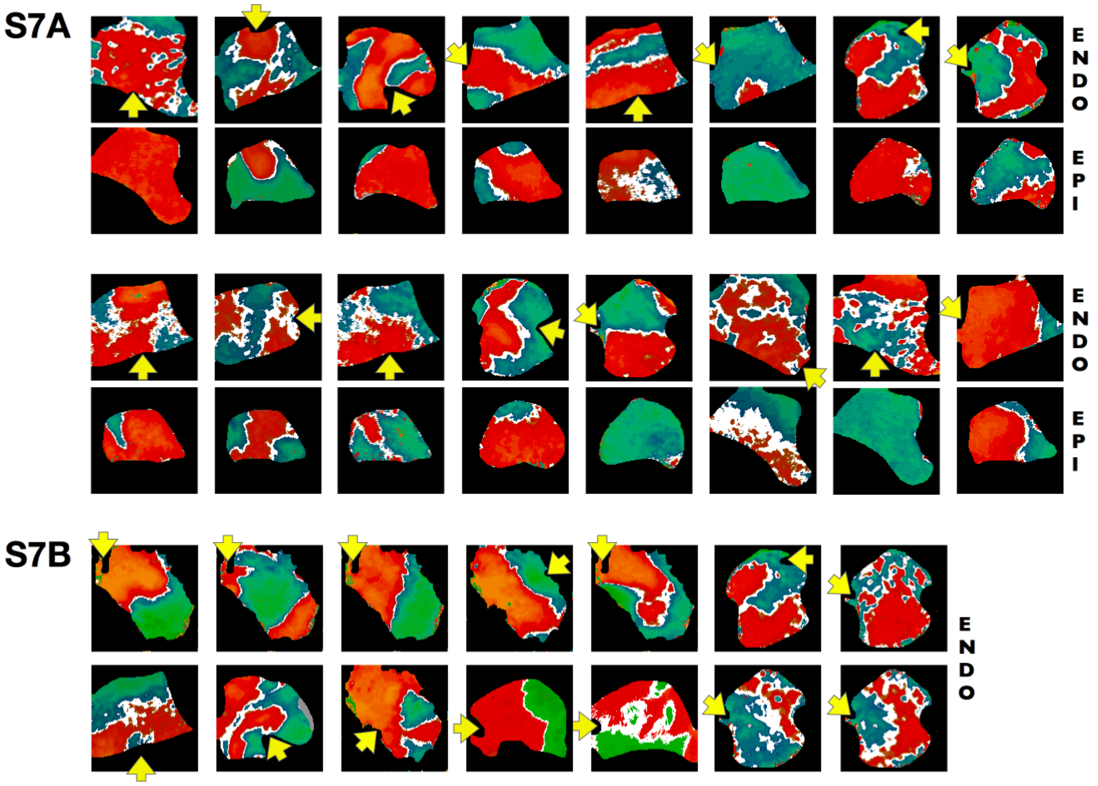

FIG. 7. **S7. Complex spatial alternans patterns.** A) Endocardium and corresponding epicardium. B) Mixed endocardial maps.

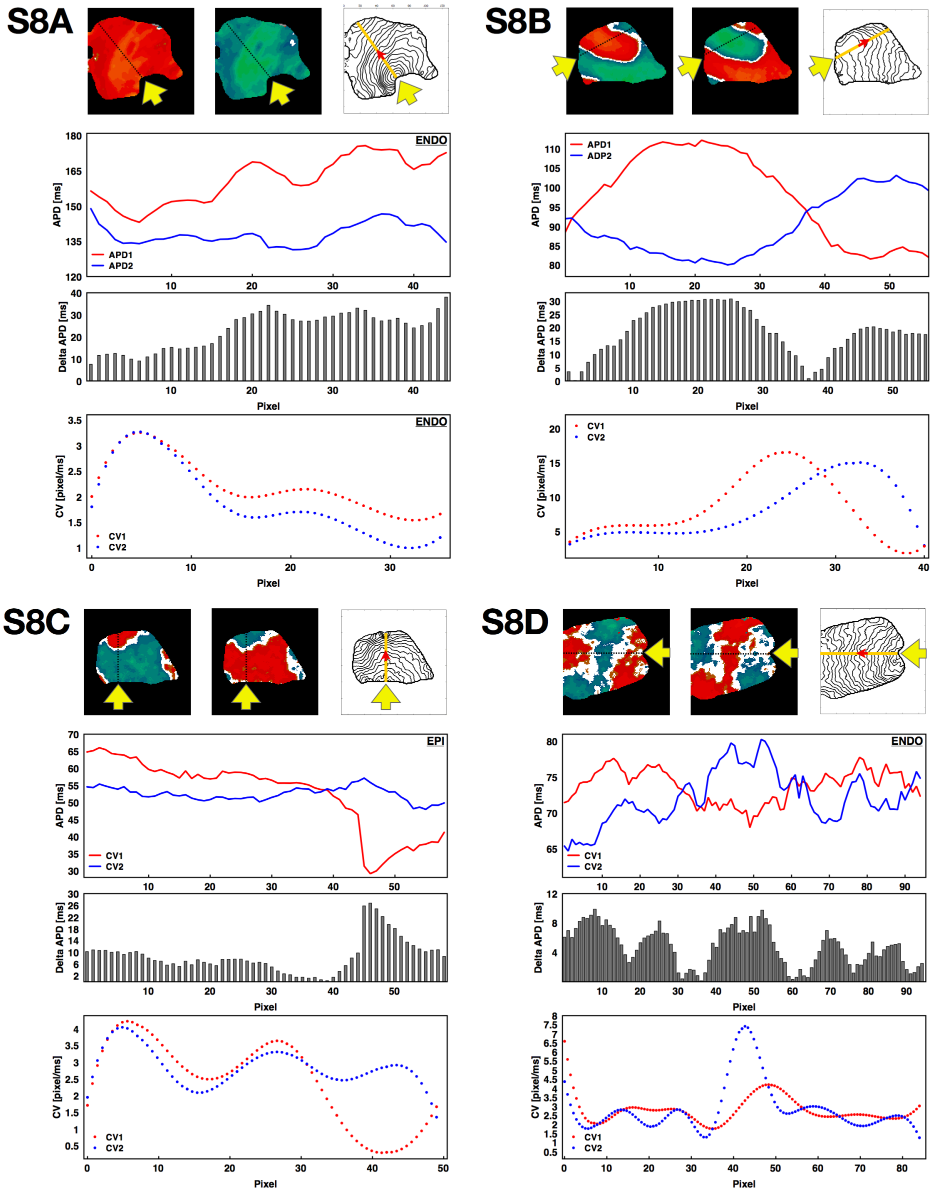

FIG. 8. **S8. Optical mapping data analysis.** Representative examples of alternans maps, isochrone maps, APD on a line, alternans amplitude and conduction velocity. The four panels show from A to D increasing spatial complexity (bottom graphs) in the direction of propagation of the action potential wave.

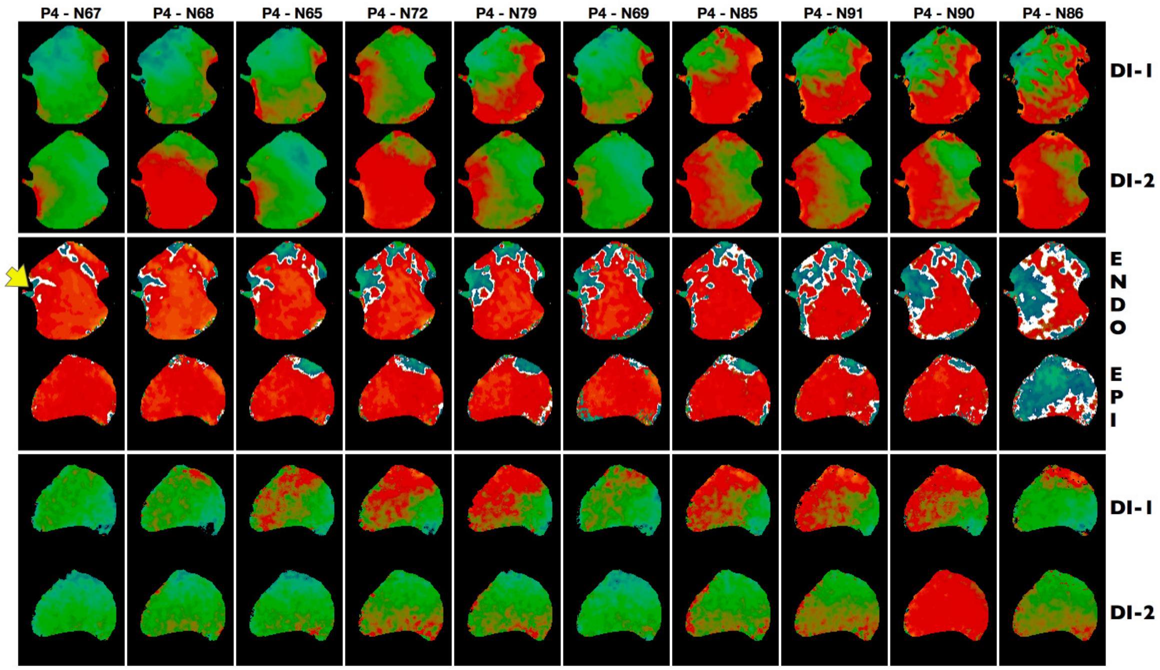

FIG. 9. **S9. Alternans patterns dependence on repolarization initial conditions.** Endocardial (top) and epicardial (bottom) alternans patterns and corresponding repolarization maps for the first two beats of the stimulation protocol in a given preparation over a 20-minute interval at a single CL of 150 ms. To facilitate comparisons, the patterns are arranged in order of similarity, but it is important to note that their appearance is not in chronological order (the experiment number is indicated on top of each panel). Pacing site is indicated by the yellow arrow.

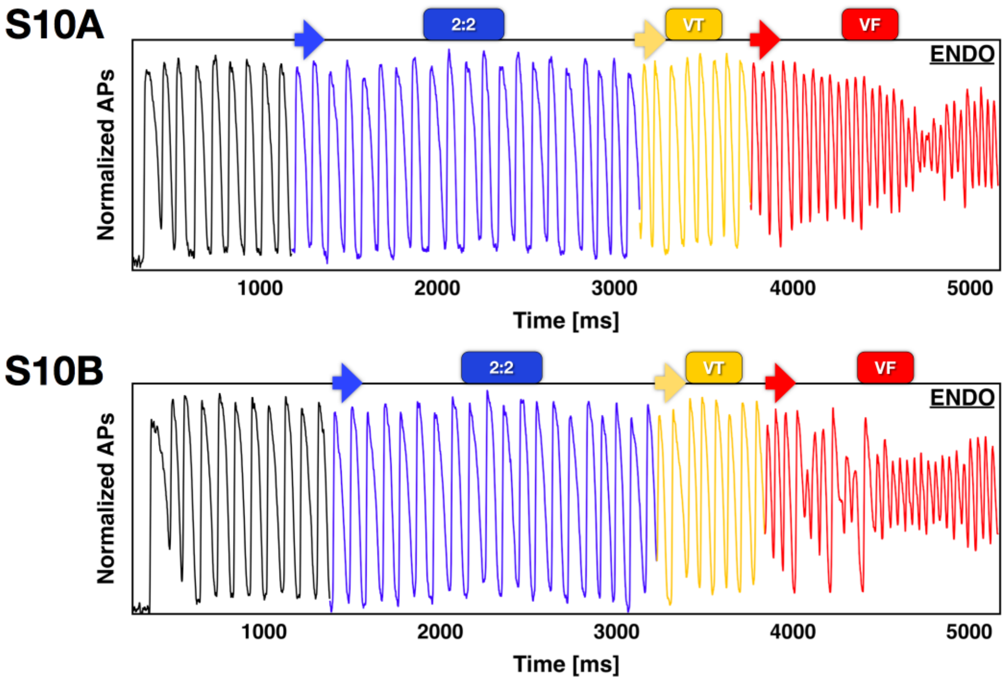

FIG. 10. **S10. Alternans development leading to ventricular fibrillation.** Representative examples of transition from normal rhythm (black) to 2 : 2 alternans (blue) to ventricular tachycardia (VT, yellow) to ventricular fibrillation (VF, red). The signals were recorded from the endocardial surface.
